# Investigating single amino acid substitutions in PIM1 kinase: A structural genomics approach

**DOI:** 10.1371/journal.pone.0258929

**Published:** 2021-10-22

**Authors:** Alaa Shafie, Shama Khan, Sagar Batra, Farah Anjum, Taj Mohammad, Shoaib Alam, Dharmendra Kumar Yadav, Asimul Islam, Md. Imtaiyaz Hassan

**Affiliations:** 1 Department of Clinical Laboratory Sciences, College of Applied Medical Sciences, Taif University, Taif, Saudi Arabia; 2 Drug Discovery and Development Centre (H3D), University of Cape Town, Rondebosch, Cape Town, South Africa; 3 Amity Institute of Biotechnology, Amity University Rajasthan, Jaipur, Rajasthan, India; 4 Centre for Interdisciplinary Research in Basic Sciences, Jamia Millia Islamia, New Delhi, India; 5 Department of Biotechnology, Jamia Millia Islamia, Jamia Nagar, New Delhi, India; 6 College of Pharmacy, Gachon University of Medicine and Science, Yeonsu-gu, Incheon City, South Korea; North-Eastern Hill University, INDIA

## Abstract

PIM1, is a serine/threonine proto-oncogene kinase, involved in many biological functions, including cell survival, proliferation, and differentiation, thus play a key role in oncogenesis. It plays a crucial role in the onset and progression of various hematopoietic and non-hematopoietic malignancies, including acute myeloid leukemia and prostate cancer. Mutations in PIM1, especially in its kinase domain, can induce abnormal structural changes and thus alter functionalities that can lead to disease progression and other complexities. Herein, we have performed an extensive analysis of the PIM1 mutations at sequence and structure level while utilizing state-of-the-art computational approaches. Based on the impact on PIM1, numerous pathogenic and destabilizing mutations were identified and subsequently analyzed in detail. Finally, two amino acid substitutions (W109C and F147C) in the kinase domain of PIM1 were selected to explore their impact on the PIM1 structure in a time evolution manner using all-atom molecular dynamics (MD) simulations for 200 ns. MD results indicate significant conformational altercations in the structure of PIM1, especially upon F147C mutation. This study provides a significant insight into the PIM1 dysfunction upon single amino acid substitutions, which can be utilized to get insights into the molecular basis of PIM1-associated disease progression.

## Introduction

The progression of cancer arises from failure at multiple cellular levels, including abnormal gene expression, metabolic conditions, abnormal signal transduction, and genetic alterations [1]. Alterations at genomic and proteomic levels cause significant changes to the structural and non-structural motifs of proteins, ultimately resulting in disease progression [2]. PIM1 is a serine/threonine kinase involved in regulating cell cycle progression and apoptosis. It has been implicated in the progression of various complex diseases, including hematopoietic and non-hematopoietic cancers, such as acute myeloid leukemia and prostate cancer [3]. PIM1 is expressed in the spleen, thymus, bone marrow, prostate, and oral epithelial and is found to be highly expressed in prostate cancer and other human malignancies [4]. It plays a central role in signal transduction involved in many biological functions, including cell survival, proliferation, and differentiation, thus play a key role in oncogenesis [3]. The PIM1 overexpression in prostate cancer has been found to decrease the patients’ survival [5].

PIM1 contains 313 amino acid residues consisting of several conserved motifs with a glycine loop motif, a phosphate-binding site, and a proton acceptor site (active site) [6]. Several natural mutations of the PIM1 have been shown to be effective in its deactivation, thermodynamically stability, altered metal binding, and altered function of the catalytic and allosteric sites [7, 8]. This altered function might lead to the onset and progression of various complex diseases, including cancer and acute myeloid leukemia [8]. Although all the mutations found in proteins are not structurally or functionally affecting, but many of them are found to be harmful to human health [9]. Only one-third of the non-synonymous (ns) mutations are appeared to be deleterious in experimental findings [10]. Compelling the prospect into consideration that PIM1 plays a vital role in cell cycle progression and apoptosis where different mutations can cause complex diseases, including cancer, we envisioned exploring the impacts of mutations on PIM1 using advanced computational approaches. Initially, we have taken a list of 185 mutations found in the whole protein where a total of 142 mutations lie in the kinase domain of PIM1 were studied in detail. Finally, two mutations, W109C and F147C present in the kinase domain of the PIM1 protein were selected and studied in detail to examine their time evolution impact using all-atom molecular dynamics (MD) simulations and essential dynamics for 200 ns.

## Materials and methods

### Retrieval of data

The UniProt [11] database was used to extract the sequence of human PIM1 protein (UniProt ID: P11309). The data in Ensembl [12], ClinVar [13], COSMIC [14], gnomAD [15], and TOPmed were used to create a list of mutations. The list was cleaned up by removing the protein truncations and duplicate variants. Furthermore, the Protein Data Bank [16] database was used to obtain a non-mutated high-resolution crystal structure of the PIM1 kinase domain (PDB ID: 1XWS). The mutations were incorporated structurally in the PIM1 coordinates using the mutagenesis wizard embedded in PyMOL software.

### Sequence-based predictions

#### SIFT

Sorting intolerant from tolerant (SIFT) is a web-based server (https://sift.bii.a-star.edu.sg/) that predicts and computes the data on the basis of sequence similarity, identity, and physico-chemical alterations between the substituted amino acids variants. It is useful to estimate if an amino acid substitution is likely to affect the protein function [17].

#### PolyPhen2

Polymorphism Phenotyping 2 (PolyPhen2) (http://genetics.bwh.harvard.edu/pph2/) is a computational tool that uses sequence homology, Pfam annotations, 3D structures to estimate the impact of an amino acid change on the structure and function of a protein. PolyPhen-2 provides a qualitative approach to score for the most damaging or least affecting variant using a set of classifier models corresponding to the HumDiv and HumVar databases [18].

#### PROVEAN

Protein Variation Effect Analyzer (PROVEAN), a web server (http://provean.jcvi.org/index.php), uses a rapid set of computational algorithms to predict the impact of amino acid substitution on a given protein sequence. First, PROVEAN analyzes the protein sequence and obtains the score by using an alignment method. Then, the score is generated by computing each value obtained from the sequence cluster [19].

#### CADD

Combined Annotation Dependent Depletion (CADD) is a computational tool (https://cadd.gs.washington.edu/) that assesses the damaging effect in the protein sequence caused by variation (insertion, deletion or substitution) of amino acids. CADD may be used in context to objectively prioritize functional, deleterious, and disease causative variations across a wide variety of functional categories, impact sizes, and genetic architectures [20].

#### REVEL

Rare Exome Variant Ensemble Learner (REVEL) is a computational method (https://redmine.igm.cumc.columbia.edu/) that exploits an ensemble approach to predict the deleterious effect caused by the advent of missense variation. REVEL utilizes a set of various computational tools such as MutPred, FATHMM v2.3, VEST 3.0, PolyPhen-2, SIFT, PROVEAN, Mutation Assessor, Mutation Taster, LRT, GERP^++^, SiPhy, phyloP, and PhastCons to compute and generate robust scores to validate the likelihood of pathogenicity in the protein sequence [21].

#### MetaLR

MetaLR (https://sites.google.com/site/jpopgen/dbNSFP) employs regression models to predict the damaging effects of missense variants by combining nine different independent variant values while estimating the allelic frequency. Variations are categorized as ’tolerated’ or ’damaging,’ indicating which variant is more likely to be harmful [22].

#### SNPMuSiC

SNPMuSiC is web-based computational software (http://dezyme.com/en/Software) used to understand the molecular impacts of variants. It compares an exclusive prediction technique on numerous independent test sets from various databases such as, DbSNP, SwissVar, and HumSaVar, which may be utilized with experimental and modeled structures. Furthermore, SNPMuSiC integrates nsSNPs variants from databases to obtain a training dataset and further classify them among “Neutral” and “Deleterious” variants [23].

### Structure-based predictions

#### MAESTRO

MAESTRO is a bioinformatics tool (http://biwww.che.sbg.ac.at/MAESTRO) that builds a multi-agent machine learning system based on protein structures. It provides expected Gibbs free energy values as well as also computes a confidence estimation for the prediction. It allows for high throughput scanning of multi-point mutations, allowing for thorough control of mutation sites and types. To predict stabilizing disulfide bonds, the program has a special mode [24]. MAESTRO’s prediction power for single point mutations and disulfide bond stabilization is comparable to any other approach.

#### CUPSAT

CUPSAT is a web-based tool (http://cupsat.tu-bs.de/) that analyzes and predicts the effect of amino acid substitution on protein stability. The software package exploits structural and conformational specificities to calculate the torsional potential between atoms [25]. In addition, this software predicts energy scores, specifically the difference in free energy of folding and unfolding structures between wild-type and mutant, to calculate the impact.

#### mCSM

mCSM is a computational program (http://biosig.unimelb.edu.au/mcsm/) that utilizes the protein residue environment used to train prediction algorithms by encoding various patterns in between their atoms to understand how mutation affects the structural conformation and stability for protein-protein and protein-nucleic acid interactions. The program is driven by analyzing the frequency of each residue in a given data set for both wild-type (WT) and mutational variant [26].

#### SDM

Site-directed mutation (SDM) is a web server (http://marid.bioc.cam.ac.uk/sdm2) based on a knowledge-based approach and predicts the difference of stability between WT and mutant protein structures. In addition, SDM calculates environment-specific substitution tables (ESSTs) and further computes the thermodynamic parameter corresponding to the folding-unfolding free energy of the protein structure [27].

#### DUET

DUET is an integrated computational web service (http://biosig.unimelb.edu.au/duet/) used for studying missense mutations in proteins using an integrated computational method. DUET combines the findings of two complementary methodologies (mCSM and SDM) in an optimized predictor utilizing Support Vector Machines (SVM), resulting in a consensus prediction.

#### DynaMut and DynaMut2

DynaMut and DynaMut2 are computational tools (http://biosig.unimelb.edu.au/dynamut/) that implement two independent and distinguished normal mode methodologies for analyzing and visualizing protein dynamics and assessing the effect of missense mutations on protein dynamics and stability due to vibrational entropy and Gibbs free energy changes [28].

#### MutPred2

MutPred2 is a computational tool (http://mutpred.mutdb.org/) that is based upon machine learning approaches. MutPred searches for molecular signals with a data collection of Mendelian disease variations and a data set of *de novo* mutations diagnosed in various diseases and estimate the proportion of damaging missense variations in the respective genome. The quantified data set is then used to priorities high-scoring variations and empirically evaluate their functional significance [29].

#### ENCoM

ENCoM (https://labworm.com/tool/encom) is a coarse-grained analysis approach that uses computational processing to account for mutations’ impact on eigenvectors eigenvalues considering atom-specific side-chain interactions. These approaches may investigate probable motions around an equilibrium conformation by computing the eigenvectors and eigenvalues associated with distinct normal modes. Each normal mode depicts the protein collectively, which is correlated, and resulting in a complicated type of motion [30].

#### Dezyme- PoPMuSiC and HoTMuSiC

Dezyme is an online platform (http://dezyme.com/en/Software) that integrates various *in-silico* prediction tools. For example, PoPMuSiC is a program of Dezyme that allows the construction of mutant proteins with regulated thermodynamic stability qualities using an *in-silico* based predictor. The experimental or modeled protein structure calculates the changes in folding free energy of a specific protein or peptide under point mutations [31]. On the contrary, HOTMuSiC is another Dezyme method that enables you to generate mutant proteins with regulated heat stability computationally. On the basis of the experimental or predicted protein structure, it measures the changes in melting temperature of a specific protein or peptide under point mutations [32].

### MD simulations

#### Systems preparation

The structural coordinates of WT PIM1 were downloaded from the PDB (PDB ID: 1XWS). The crystallographic water molecules were deleted, and missing hydrogen atoms were added along with the allocation of appropriate charge at neutral pH. The pre-processing and energy minimization of the structure was done by the Protein Preparation Wizard of the Schrödinger suite [33]. The mutagenesis wizard of PyMOL was utilized to induce mutations in the protein structure [34]. All the structures were further imperiled to energy minimization using the OPLS-2005 force-field [35] with a root mean square deviation (RMSD) cut-off value of 0.30 Å to minimize all possible steric clashes between the residues.

#### Simulations protocol

All-atom MD simulations were run on a GPU accelerated engine PMEMD encoded in the Amber 18 package for 200 ns [36]. Amber 18 forcefield FF14SB was applied on all three systems during the simulation [37]. The solvation was carried out in a TIP3P solvent model [38] inside a 10 Å box edge using the LEaP module. Then, an appropriate number of counterions (Na^+^ and Cl^-^) was added to each system for neutralization prior to the minimization phase. A partial minimization for 1500 steps was achieved with a 500 kcal/mol restraint potential gradient. Later, a full minimization for 1000 steps was done using the conjugate gradient approach by removing all applied restraints on the partial minimization step. After, all systems were gradually heated from 0 K to 300 K, allowing a constant volume and number of atoms for each system. Subsequently, equilibration was carried out by retaining the 500 ps equilibration step, ensuring a constant temperature of 300 K. NPT (isobaric-isothermal ensemble) was endorsed to maintain a constant pressure of 1 bar and number of atoms within each system. Finally, the MD trajectories for 200 ns of each system were produced from the production phase by integrating the SHAKE algorithm [39].

#### Post-dynamic trajectories analysis

The structural coordinates of all three systems were saved for every 1 ps, and the trajectory curves were calculated through the CPPTRAJ module [40] of Amber 18. The RMSD, RMSF, *R*_g_, SASA, intramolecular hydrogen bonding, secondary structure analysis, distance correlation matrix, and PCA of all three systems were calculated. Origin [41] and VMD [42] tools were used for generating plots and analyses of MD results.

### Dynamics of the cross-correlation matrix

The DCCM analysis was carried out to determine the co-ordinal deviations and behaviors in C_α_ atoms of PIM1 and its variants. Factors for i and j cross-correlation C_α_ atoms can be described as:

$$C_{ij}=\frac{<\Delta ri.\Delta rj>}{{\left(<{\Delta r}_{i}^{2}><{\Delta r}_{j}^{2}>\right)}^{\frac{1}{2}}}$$ (1)

where Δr_i,j_ is the movement of i^th^ and j^th^ atom average point. Correlated movements are signified by C_ij_ = 1; however, C_ij_ = -1 is supposed to be highly anti-correlated. The deviation of atomic movements from 1 to -1 describes that i and j movements are correlated and anti-correlated.

### Principal component analysis

PCA is a useful approach to examine a protein molecule’s conformational movements and folding behavior by illustrating its atomic movements [43]. PCA generates atomic movements from MD trajectories by retaining dimensional reduction [44, 45]. The PCA was carried out using the covariance matrix C, based on the atomic coordinates and corresponding eigenvectors (EVs) [46]. The positional covariance matrix C was constructed through the following equation:

$$C_{i}=<(q_{i}-<q_{i}>)(q_{j}-<q_{j}>)>\;(i,j=1,2,\ldots ,3N)$$ (2)

where q_i_ and q_j_ signify the cartesian coordinates for the i^th^, j^th^ position of C_α_ atom and N is the number of C_α_ atoms.

## Result and discussion

The mutations of the PIM1 kinase domain were extracted from the Ensembl database [47]. A total of 4,588 mutations were recognized in the PIM1, including post-translational variations, missense variations, exonic variations, and intronic variations. According to the database, only 142 mutations were found in the kinase domain of PIM1. However, the center of our study aims to find the impacts of the selected variations on the structure and function of the PIM1.

### Sequence- and structure-based analysis

**Fig 1:**
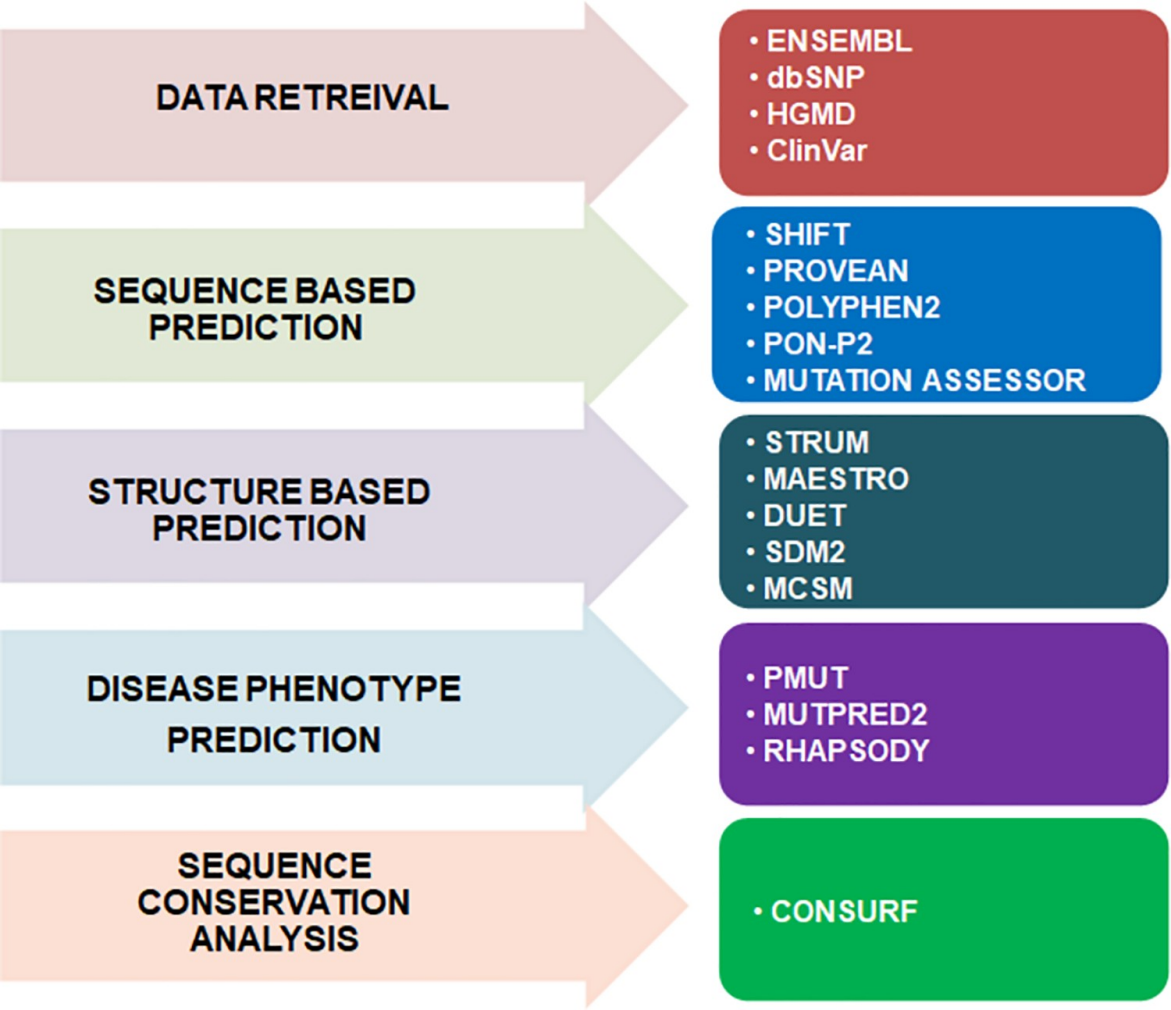
Overview of the methods used to predict the pathogenic mutations and their impact on PIM1 at the sequence, structure, and function levels.

The extracted mutations were diagnosed in a serial manner consisting of various steps [48, 49]. Primarily the high-scoring mutations of the PIM1 gene were categorized into sequence-based and structure-based analyses. All the mapped mutations of PIM1 were first exposed to several computational tools such as SIFT, Polyphen2, PROVEAN, CADD, REVEL, MetaLR, and SNPMuSiC and predicted damaging or non-damaging variants by calculating their respective cut-off values. Further, the mutational interpretation was followed by the structure-based stability prediction using a variety of machine-learning-based web servers, MAESTRO, CUPSAT, mCSM, SDM, DUET, DynaMut, DynaMut2, ENCoM, MutPred2, PoPMuSiC, and HoTMuSiC. A basic outline of the tools used in this study is illustrated in **Fig 1**.

Sequence-based tools quantify deleterious and neutral variants among the selected list of variations. For example, where, SIFT algorithm predicts the tolerance score (tolerated = <0.05) of substituted residues, PolyPhen2 classifies its prediction scores into three classes, i.e., benign (score <0.2), possibly damaging (score >0.2 and <0.96), and probably damaging (score >0.96), PROVEAN webserver was used to differentiate the deleterious residues from neutral ones (score >-2.5) on a cut-off scale whereas, REVEL calculates a cumulative score and projects neutral (score <0.5) and disease type (score >0.5) for each variant. Furthermore, MetaLR classifies the variants in a range of 0 to 1, where 1 is the most deleterious. Taking the analogy further, CADD applies the strictest criterion among all the sequence-based predictors and provides a cut-off score of 30, above which CADD highly recommends the variant to be deleterious.

Thermodynamics is an essential measure of determining protein structure stability [50]. Disturbance in the structure and function are few known effects to be generated by the cause of mutations. However, establishing the difference in both the protein states (folded and unfolded) in terms of free energy Δ*G* could be a governing method deciding the structural stability of the protein (a). The certainty for the WT and mutant structure of the same species having a significant change in their Δ*G* energies is high; therefore, it is settled by calculating the ΔΔ*G* of both the structures (b). The calculation was given by the following formula.


ΔΔG=ΔGunfoldedstructure–ΔGfoldedstructure (a)

ΔΔG=ΔGmutatedstructure–ΔGwildtypestructure (b)

Using these mathematical determinants, we have predicted the stabilizing and destabilizing amino acid substitutions associated with the PIM1 kinase domain. A total of 11 novel web-based predictors were used to differentiate the stability criteria, specifically, MAESTRO, CUPSAT, mCSM, SDM, DUET, DynaMut, DynaMut2, ENCoM, MutPred2, PoPMuSiC, and HoTMuSiC. These biophysical methods used 3D PDB coordinate file as an input and were based on machine learning algorithms and support vector machines to calculate the intrinsic variability of a protein that has been triggered by mutations.

**Fig 2:**
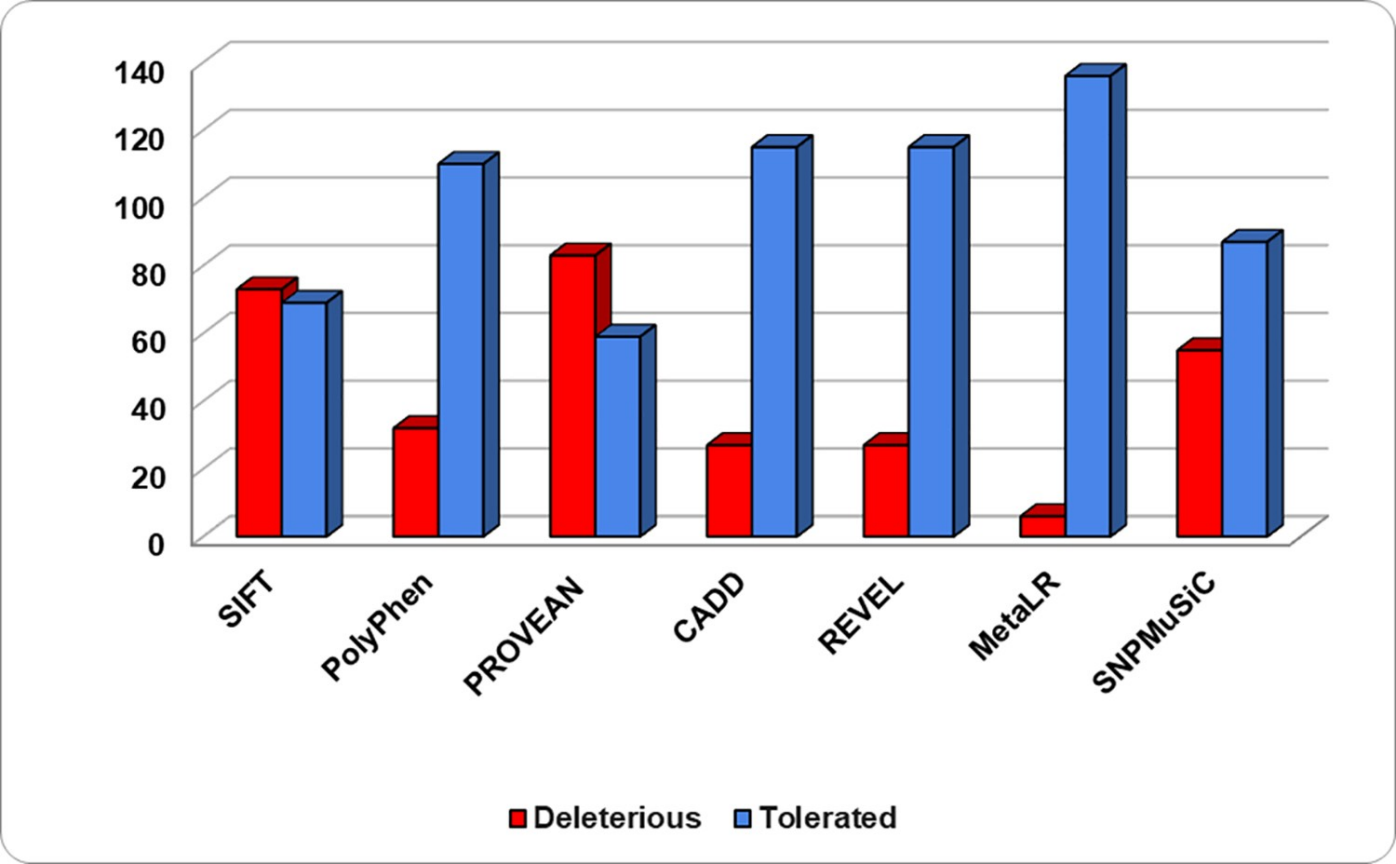
Scattering of deleterious variations in PIM1 predicted through sequence-based tools.

The extracted missense mutations of the kinase domain of the PIM1 were first executed through sequence-based predictors, and the results were successfully generated (**S1 Table**). The outcome of the sequence-based predictors showed the deleterious and tolerated substitutions, in which, SIFT-73 (51.4%), PolyPhen-32 (22.5%), PROVEAN-83(58.4%), CADD-27 (19.01%), REVEL-27 (19.01%), MetaLR-6 (4.2%), and SNPMuSiC-55 (38.7%) (out of 142 variants) accounted for deleterious substitutions (**Fig 2**).

**Fig 3:**
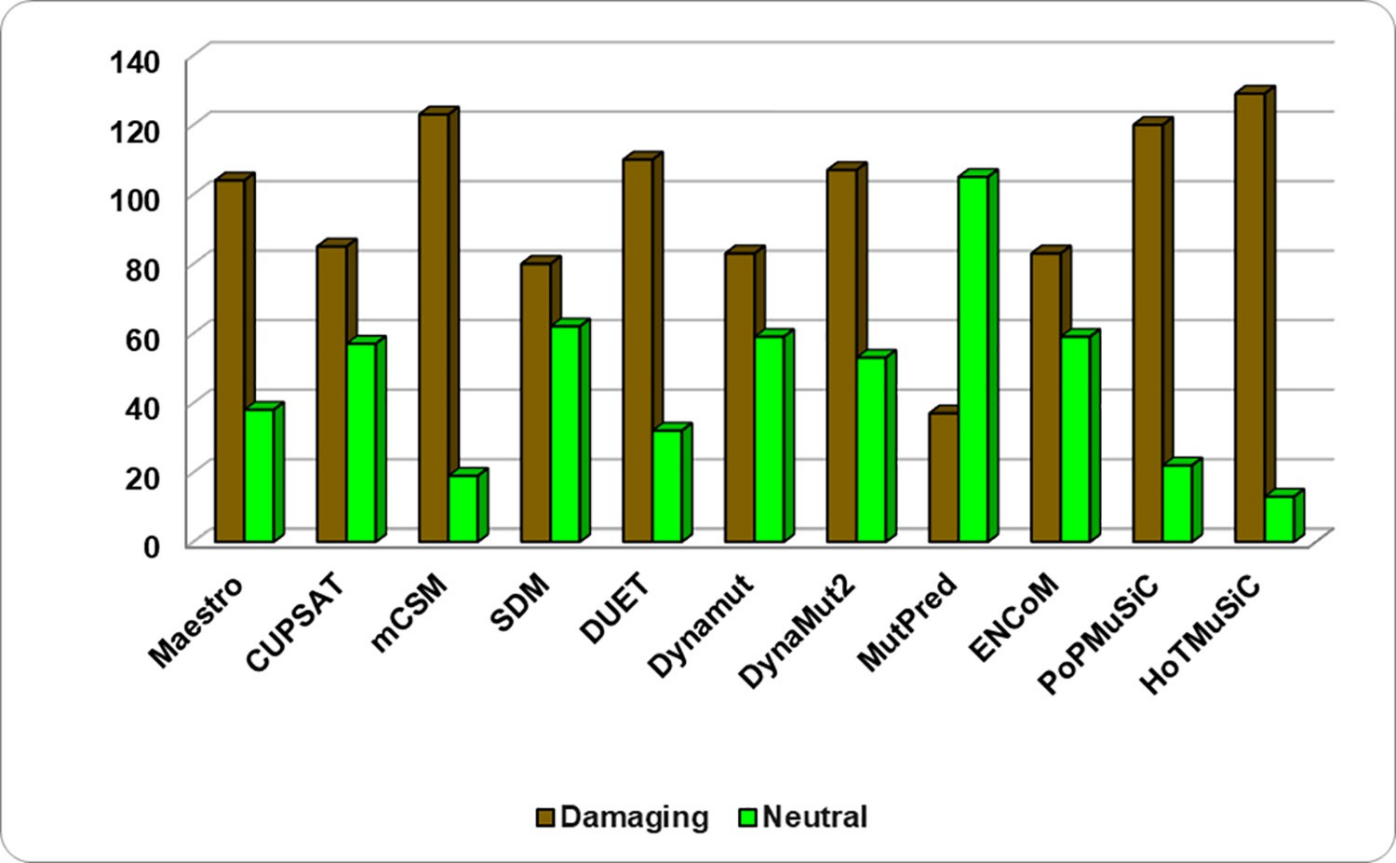
Scattering of deleterious variations in PIM1 predicted by structure-based tools.

In the structure-based predictors, a total of 142 substitutions were run into different webservers (**S2 Table**) where MAESTRO-104 (73.2%), CUPSAT-85 (59.8%), mCSM-123 (86.6%), SDM-80 (56.3%), DUET-110 (77.4%), DynaMut-83 (58.4%), Dynamut2-107 (75.3%), ENCoM-83 (58.4%), MutPred2-37 (26.05%), PoPMuSiC-120 (84.5%) and HoTMuSiC-129 (90.8%) substitutions were predicted to be destabilizing the protein structure (**Fig 3**). According to the results, the data show 26.05% deleterious substitutions from sequence-based predictions of PIM1 when considered from at least 4 predictors out of 7. Also, the structure-based analysis showed the presence of destabilizing variants by 64.7% from the kinase domain of PIM1 when confirmed from 6 or more predictors out of 11. A total of 142 collective damaging and destabilizing substitutions were obtained from both sequence-based and structure-based predictions.

### Identification of PIM1 associated functional significance

We went on to look at the structurally destabilizing variants to see how we could predict their phenotypic expression when they were mutated. MutPred tool and resulted in 142 high scoring mutations and generated values representing stabilization and destabilization. MutPred predicted values resulting in >0.5 were regarded as destabilizing mutations; however, for 142 high-confidence scoring mutations, MutPred resulted in 37 (26.05%) destabilizing mutations and disease-associated mutations.

**Fig 4:**
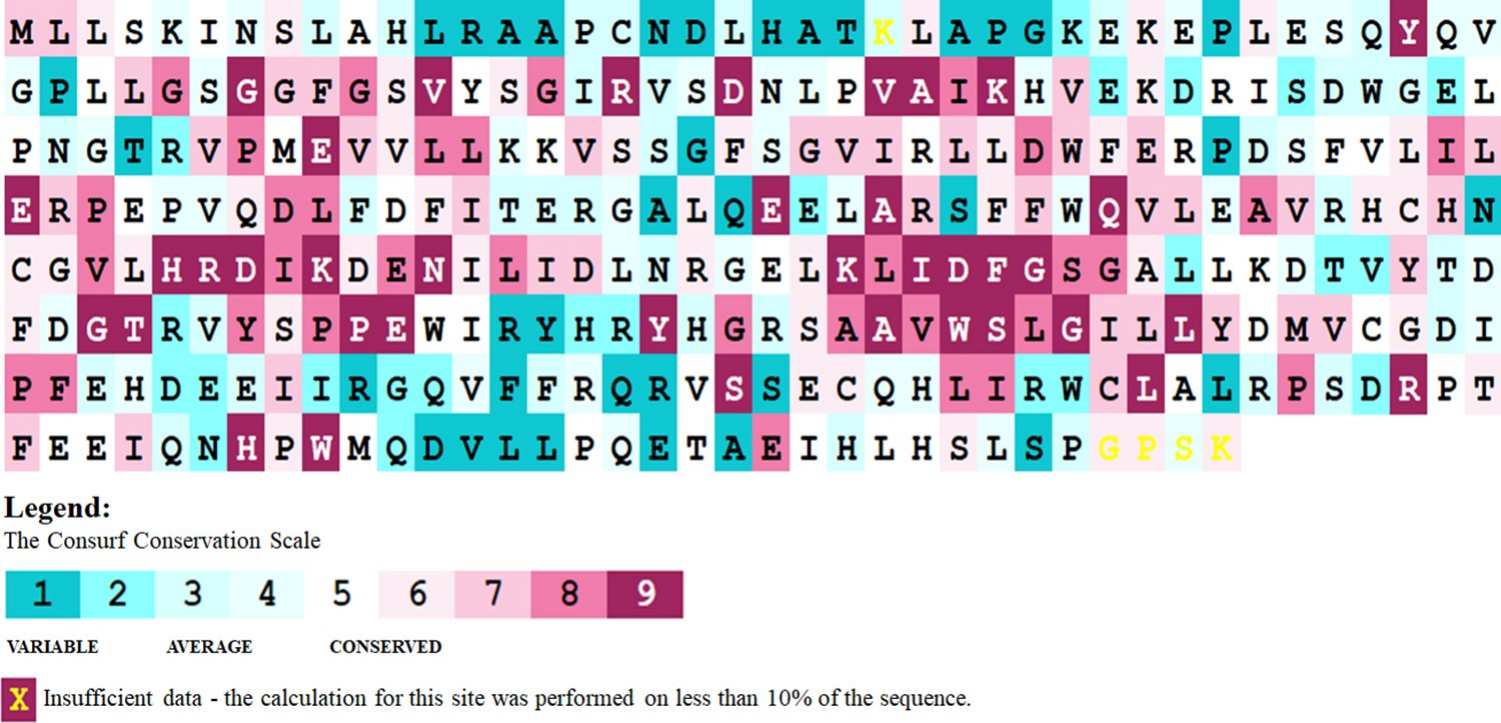
Sequence conservation analysis of PIM1 protein using ConSurf web server.

The conservation analysis of amino acid residues in a protein helps in understanding their importance and localized evolution over generations. Here, the Consurf webserver was used to do a residual conservation study, which suggests the evolutionary sustainability of the residue throughout the course. The scoring was to find the most conserved residue undergoing residual substitution and causing mutation [51]. The fact that a modification in a conserved residue causes structural and functional dysfunction of the protein is well known. As a result, in order to accept the statement, our study links the results to the mutagenic effects of residues over the protein structure. The Consurf analysis exhibited the arrangement of residues along with their conservation scores (**Fig 4**). The ConSurf analysis showed that many residues, including W109 and F147, are relatively conserved in PIM1 protein, which suggests that any mutation at these sites might affect PIM1 significantly. Using this tool, we have also calculated the possible physicochemical characteristics involving distance and charge potential required for bond formation and stabilization.

Two mutations at the C-terminal region, namely, W109C and F147C, were found out to be causing severe structural damage by intentionally replacing buried charge and resulting in H-bond breakage specifically. Finally, W109C and F147C were the most promising determinants detected, which may be involved in structural consequences of the protein malfunctioning, and were further studied in all-atom MD simulations.

### Post-dynamics trajectories analysis

**Fig 5:**
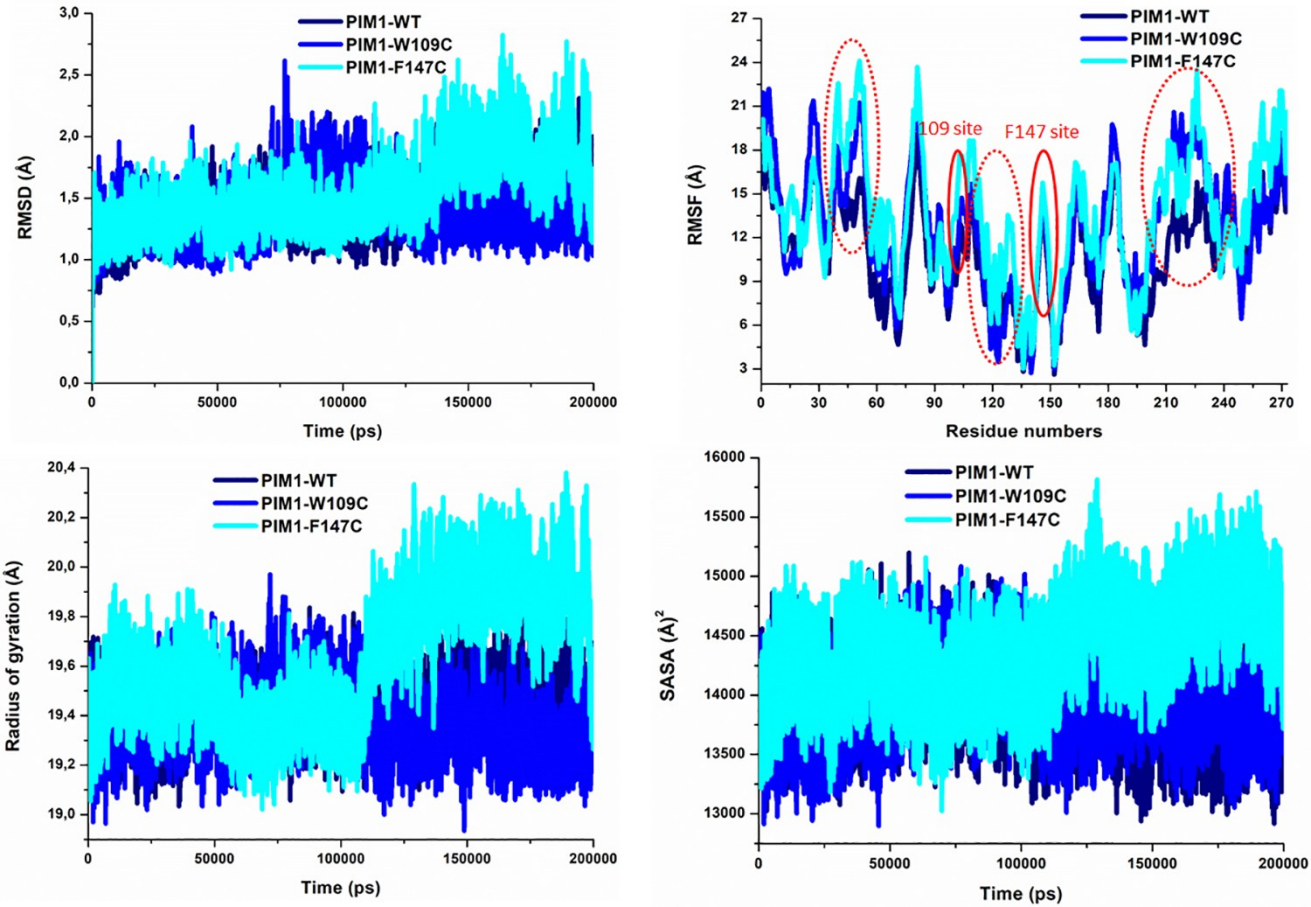
Structural dynamics of PIM1 and its mutants W109C and F147C. (**A**) RMSD, (**B**) RMSF, (**C**) *Rg*, and (**D**) SASA values across C_α_ of all three systems calculated from the MD trajectories of 200 ns. Red circles show significant fluctuations in the variants.

The conformational fluctuations and structural deviations in a protein are connected with its inherent behavior [52]. The changes in the structural integrity of a protein can induce major impacts on its activity [53]. Hence it is important to study the conformational dynamics of proteins to get deeper insights into the mutation-induced structural alterations [44, 54]. The time evolution of RMSD of C_α_ atoms was studied to ascertain the structural integrity of the simulated systems. The RMSD plot shown in **Fig 5A** suggested that all the systems attained convergence after 60 ns of the simulation. PIM1-WT showed the least deviation, whereas F147C disclosed a higher RMSD followed by W109C. PIM1 showed the highest fluctuations throughout the simulation after getting mutation at F147C and deviated from the RMSD pattern of PIM1-WT. The analysis suggests that PIM1-WT and W109C exhibited the least deviation of C_α_ atoms as compared to F147C, signifying that mutation of Phe to Cys imposed lesser stability to PIM1 structure. No significant difference was observed in the structural snaps except the loop regions of superimposed PIM1-WT, W109C, and F147C at every 50ns during the simulation (**S1 Fig**). Interestingly, the loss of some helical structures was observed in both mutants in the early simulation phase. Here, loops become more flexible with increased dynamics during the simulation most effectively in F147C, especially.

Flexibility and rigidity of amino acid residues in a protein structure accountable for various conformational changes thus functionalities of that protein [55]. Thus, the changes in the residual fluctuations in PIM1 upon mutations can be examined by studying the RMSF values [49, 56]. To explore the changes in the residual flexibility PIM1 and its mutants, RMSF of their C_α_ atoms were calculated and plotted from the MD trajectories (**Fig 5B**). The plot shows that WT PIM1 has the least fluctuations of the amino acid residues when comparing to the mutated systems. However, both mutants showed higher fluctuations among the amino acid residues during the simulation. A relatively higher pattern of fluctuations, especially in the case of F147C, was observed from the simulated trajectory. The RMSF plots showed higher fluctuation at the places of variant sites and their neighboring regions. The mutations cause higher dynamics and internal disturbances in the neighbouring areas of the variation sites which are reflecting as increased/decreased fluctuations in PIM1. The RMSF pattern can be correlated with the RMSD distribution, where higher fluctuations were seen in mutant systems. The major changes in the residual fluctuations of the mutants could be associated with the structural inactivation of PIM1.

The overall conformational changes and folding behavior of the PIM1 structure before and after inducing mutations were assessed by examining the *R*g values of all three systems. The time evolution of the *R*g values reveals the mechanism of structural compactness, stability, and folding of a protein structure [57]. We have calculated and plotted the *R*g values of PIM1-WT, W109C and F147C systems from the MD trajectories (**Fig 5C**). The *R*g of F147C exhibited the most deviation compared to WT and W109C, especially after 100 ns. The F147C structure seems to be unfolded after 110 ns of the simulation time. Overall, the *R*g evaluation of PIM1 showed reduced stability of the mutants with increased flexibility and less compactness.

The SASA was also estimated to examine the exposer of hydrophobic and hydrophilic amino acid residues present in the PIM1 structure [58]. The SASA values for PIM1-WT, W109C, and F147C were calculated and plotted over 200 ns of MD simulation (**Fig 5D**). SASA values of all three systems are shown to have a corresponding pattern of distribution as the *Rg* plot. The buried residues of the F147C system seem to be exposed to the solvent during the simulation. The plot showed that the SASA of F147C gets more exposer after 110 ns, suggestive of unfolding the protein structure. W109C mutant also exhibited more SASA values in contrast to the WT system of PIM1. The significant variation in the SASA values of F147C signified the unfolding nature of PIM1 structure upon mutation.

### Intramolecular hydrogen bond analysis

**Fig 6:**
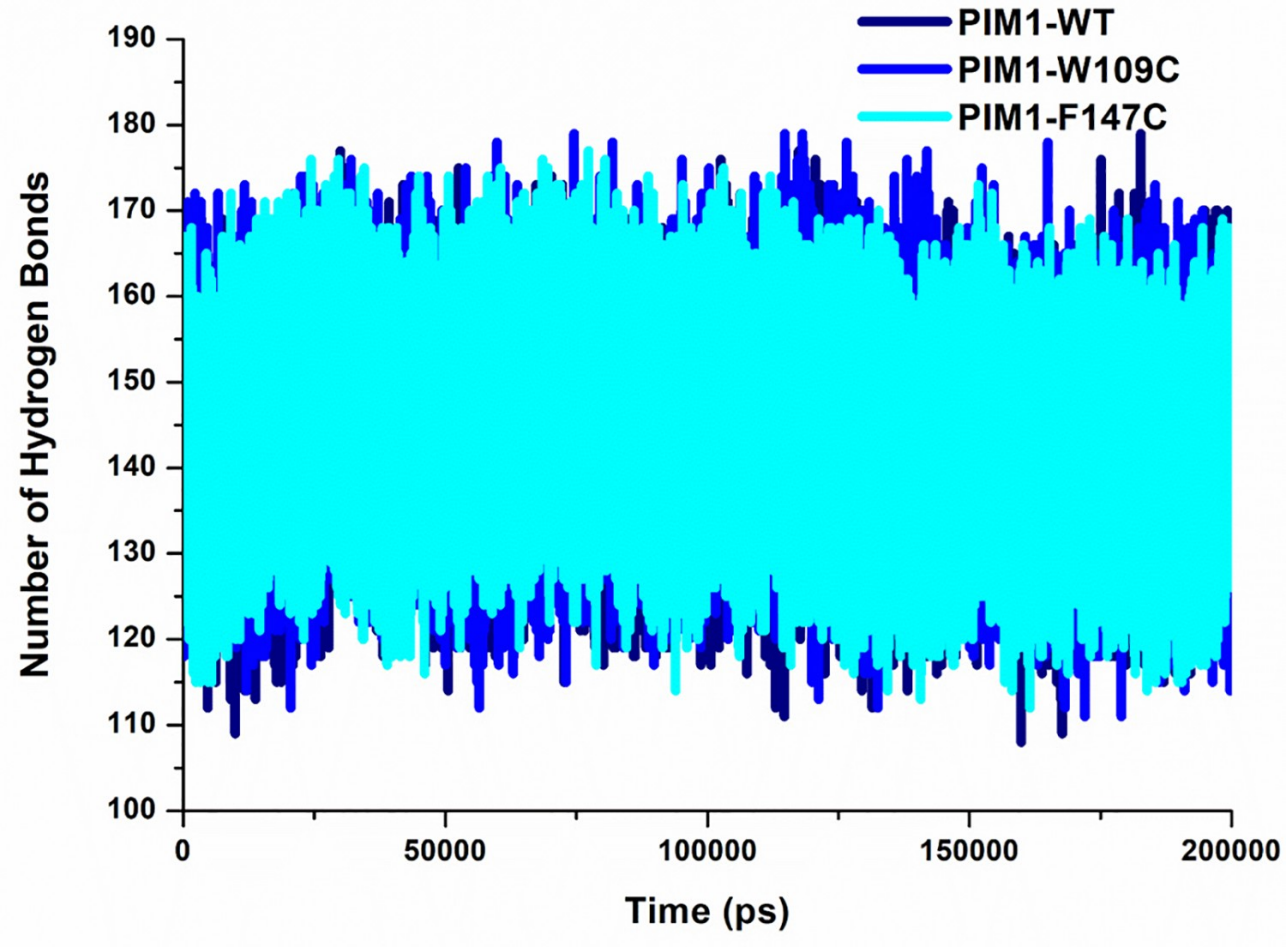
Intramolecular hydrogen bonding in PIM1 WT, W109C and F147C calculated after 200 ns MD simulation.

Intramolecular hydrogen bonding is an essential conformational shape and structural integrity of proteins [59]. Time evaluation of intramolecular hydrogen bonding is helpful to assess the structural integrity of protein structures over simulations [60]. To examine the impact of mutations on PIM1 structure, intramolecular hydrogen bond analysis was carried out and plotted concerning time (**Fig 6**). The plot showed that there was not much change in the average number of intramolecular hydrogen bonds in PIM1 before and after inducing mutations. A minor decrement of hydrogen bonding, especially in the F147C system, signified its less compactness during the simulation. The results followed an almost similar pattern of trajectories where WT and W109C systems were more stable and compact than F147C mutant.

### Secondary structure analysis

**Fig 7:**
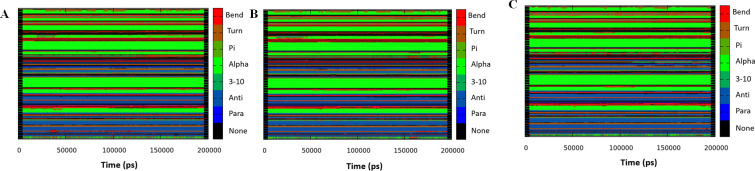
Secondary structural contents of (**A**) Wild type PIM1; (**B**) W109C; and (**C**) F147C mutants calculated after 200 ns of MD trajectories.

To examine the effect of mutations on the structural contents in PIM1 during the simulations, the time evolution of the secondary structure of all three systems was explored (**Fig 7**). As calculated from the study, the average secondary structural contents are less F147C than the WT and W109C. A significant decrease was seen in the α-helix of PIM1 after getting the F147C mutation. The residual content forming the β-strands of PIM1 is also slightly decreased in F147C, revealing its loss structure and native function. It is important to note that changes in the secondary structure of PIM1 after getting mutations elucidates less compactness of the structure due to the loop formation. The evaluation of the secondary structure content of PIM1 and its variants during the simulations would offer deeper insights into the conformational dynamics of PIM1.

### Distance correlation matrix

**Fig 8:**
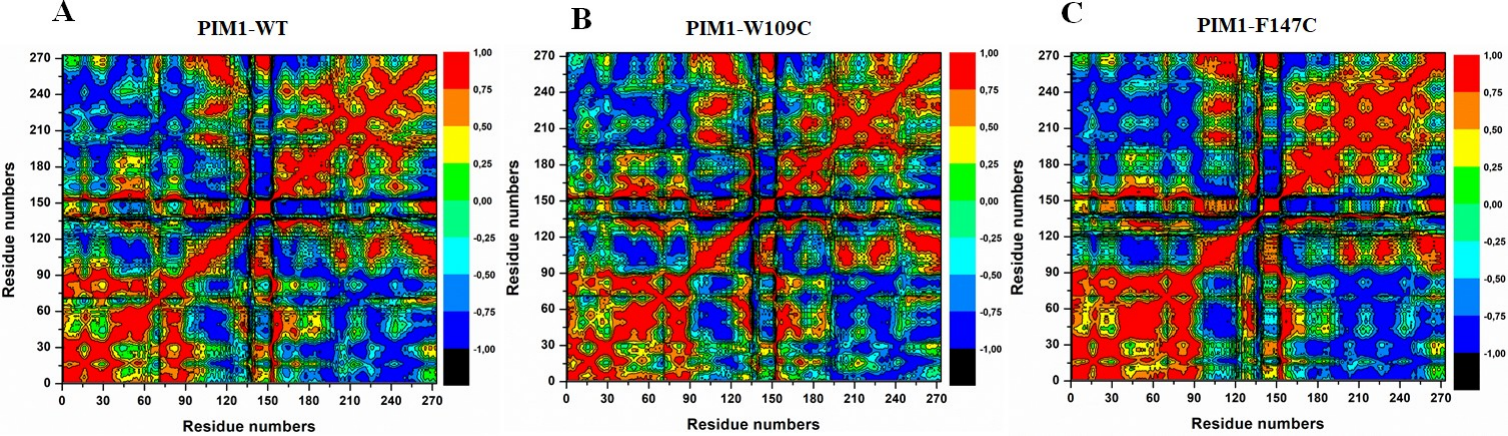
Dynamics cross-correlation matrix of (**A**) Wild type PIM1; (**B**) W109C; and (**C**) F147C mutants calculated after 200 ns of MD trajectories.

Distinctive dynamics of PIM1-WT, W109C, and F147C mutants were explored by plotting DCCM for the correlated and anti-correlated movements in the protein structure (**Fig 8**). It was observed from the plots that PIM1 was scattered into several communities across positive and negative correlations between the residual movements. The movements in PIM1-WT were seen to be equal to positive and negative movements during the simulation (**Fig 8A**). Whereas, a significant difference was seen in the case of mutants, especially in F147C, with a more positive correlation (**Fig 8C**). However, a slight negative correlation was in the W109C mutant, majorly between 150 to 250 amino acid residues (**Fig 8B**). Thus, W109C and WT PIM1 seem to be more similar when compared to F147C, suggested its decreased activity after getting mutation.

### Principal component analysis

**Fig 9:**
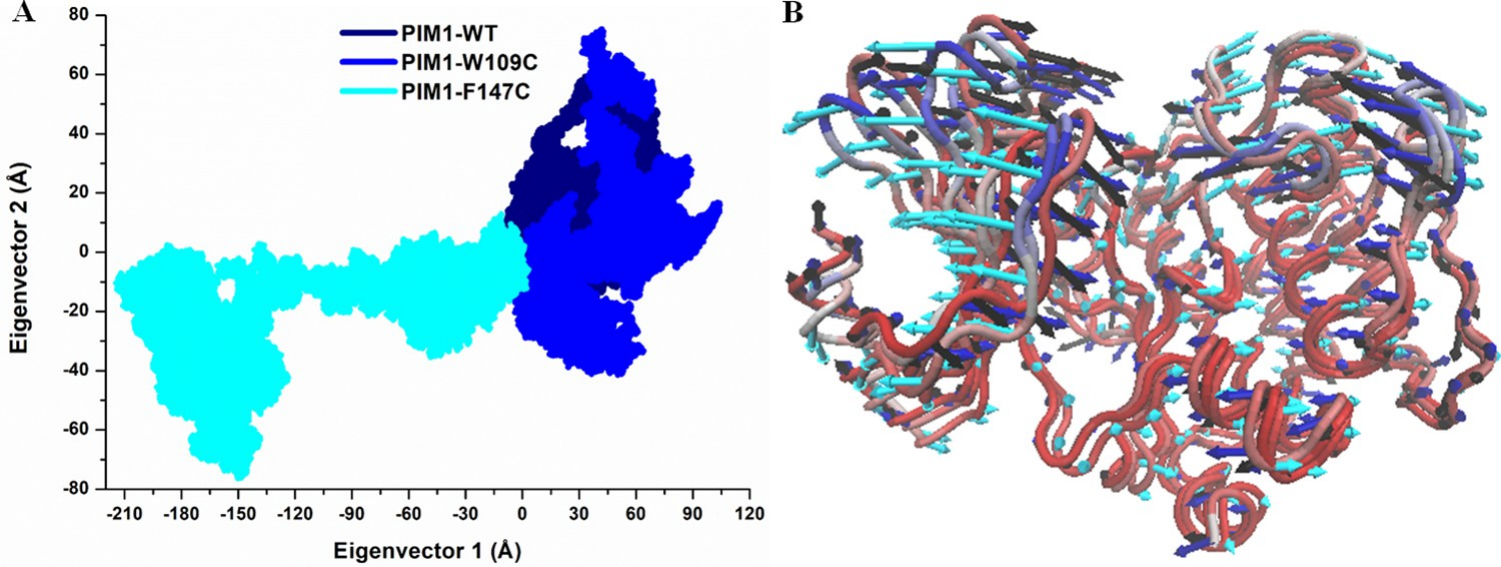
Principal component analysis for (**A**) PIM1-WT, W109C and F147C mutants. (**B**) PC1 collective motions for the obtained predominant EVs using PCA over the 200 ns MD trajectories.

To qualitatively investigate the impact of mutations on the major conformational movements in PIM1, the PCA was carried out using the principal component (PCs) based on the first two eigenvectors (EVs) (**Fig 9**). The 2D scatter plot illustrated the conformational movements occupied by PIM1 and its variants (**Fig 9A**). While the PC1 collective motions for the major EVs using the PC in PIM1 and its mutants are portrayed in **Fig 9B**. The 2D scatter plots of all three systems in **Fig 9A** signify a notable change in the overall movements of PIM1 after getting the mutations especially, F147C. As can be seen from **Fig 9A**, F147C spans a negatively correlated conformational space along EV1 (i.e., -210 Å to +05 Å) and (-75 Å to +10 Å) along with EV2. Whereas W109C showed a restricted exploration along both the eigenvectors (EV1: -5 Å to +105 Å, and PC2: -10 Å to +75 Å) compared to the WT protein. It is evident from the plot that the F147C mutant gets high negatively correlated fluctuation on EV1, signifying its altered movements. Whereas in the W109C system, the noticeable positively correlated movement on both EVs. However, PIM1-WT and W109C followed a somehow similar pattern of movements and overlapped with each other. The scattered features in **Fig 9B** represent the transition of the protein movements among the states where F147C showed higher transition compared to W109C and WT PIM1. Therefore, the results showed that F147C mutation induces significant fluctuations in the PIM1 dynamics during the simulation.

## Conclusion

To recap the study, we would like to conclude that the computational analysis projected on the kinase domain of PIM1 predicted the impact of amino acid substitutions, which are directly associated with causing the structural and conformational destabilization of the protein. Here, variant W109C and F147C were predicted to significantly alter the metal-binding followed by the loss of the allosteric site at R105 and K169, respectively. However, variant F147C promoted a catalytic site gain at the K169 position. At last, the examinations of these variants were carried out, especially with the help of MD simulation studies for 200ns. The variation W109C [61] and F147C [62] also correlate with the previously reported data. The MD analyses have shown that the pathogenic impact of these mutations may occur due to the caused structural alterations in PIM1. MD simulation analyses, including RMSD, RMSF, *Rg*, SASA, and PCA, indicate that PIM1 suffers a significant conformational loss in its structure due to mutations, especially in the case of F147C. Thus, the study provides deeper insights into the conformational behavior of PIM1 upon mutations. These insights may be further explored to develop therapeutic strategies against PIM1 associated diseases, including cancer.

## Supporting information

S1 Fig Structural snapshots of PIM1 (**A**) WT, (**B**) F147C, and (**C**) W109C at an interval of 50 ns from 0 to 200 ns of simulation. Corresponding panels show the RMSD of structural alignment of PIM1 at different times. Structures were drawn using PyMOL (https://pymol.org/2).
(DOCX)

S1 Table Sequence based prediction of mutations associated with PIM1 kinase domain. (DOCX)

S2 Table Structure based prediction of mutations associated with PIM1 kinase domain. (DOCX)

## References

[1] Sekido Y. Genomic abnormalities and signal transduction dysregulation in malignant mesothelioma cells, Cancer science 2010;101:1–6. doi: 10.1111/j.1349-7006.2009.01336.x 19793348 PMC11159396

[2] Baak J, Path F, Hermsen M et al. Genomics and proteomics in cancer, European journal of cancer 2003;39:1199–1215. doi: 10.1016/s0959-8049(03)00265-x 12763207

[3] Merkel AL, Meggers E, Ocker M. PIM1 kinase as a target for cancer therapy, Expert opinion on investigational drugs 2012;21:425–436. doi: 10.1517/13543784.2012.668527 22385334

[4] Bachmann M, Möröy T. The serine/threonine kinase Pim-1, The international journal of biochemistry & cell biology 2005;37:726–730. 15694833 10.1016/j.biocel.2004.11.005

[5] Luszczak S, Kumar C, Sathyadevan VK et al. PIM kinase inhibition: Co-targeted therapeutic approaches in prostate cancer, Signal Transduction and Targeted Therapy 2020;5:1–10. doi: 10.1038/s41392-019-0089-y 32296034 PMC6992635

[6] Arunesh GM, Shanthi E, Krishna MH et al. Small molecule inhibitors of PIM1 kinase: July 2009 to February 2013 patent update, Expert opinion on therapeutic patents 2014;24:5–17. doi: 10.1517/13543776.2014.848196 24131033

[7] Lori C, Lantella A, Pasquo A et al. Effect of single amino acid substitution observed in cancer on Pim-1 kinase thermodynamic stability and structure, PloS one 2013;8:e64824. doi: 10.1371/journal.pone.0064824 23755147 PMC3673989

[8] Shah N, Pang B, Yeoh K-G et al. Potential roles for the PIM1 kinase in human cancer–a molecular and therapeutic appraisal, European journal of cancer 2008;44:2144–2151. doi: 10.1016/j.ejca.2008.06.044 18715779

[9] Kucukkal TG, Petukh M, Li L et al. Structural and physico-chemical effects of disease and non-disease nsSNPs on proteins, Current opinion in structural biology 2015;32:18–24. doi: 10.1016/j.sbi.2015.01.003 25658850 PMC4511717

[10] Bromberg Y, Rost B. Correlating protein function and stability through the analysis of single amino acid substitutions, BMC bioinformatics 2009;10:1–9. doi: 10.1186/1471-2105-10-1 19758472 PMC2745590

[11] Consortium U. UniProt: a hub for protein information, Nucleic acids research 2015;43:D204–D212. doi: 10.1093/nar/gku989 25348405 PMC4384041

[12] Hubbard T, Barker D, Birney E et al. The Ensembl genome database project, Nucleic Acids Research 2002;30:38–41. doi: 10.1093/nar/30.1.38 11752248 PMC99161

[13] Landrum MJ, Lee JM, Benson M et al. ClinVar: public archive of interpretations of clinically relevant variants, Nucleic acids research 2016;44:D862–D868. doi: 10.1093/nar/gkv1222 26582918 PMC4702865

[14] Tate JG, Bamford S, Jubb HC et al. COSMIC: the catalogue of somatic mutations in cancer, Nucleic acids research 2019;47:D941–D947. doi: 10.1093/nar/gky1015 30371878 PMC6323903

[15] Karczewski K, Francioli L. The genome aggregation database (gnomAD), MacArthur Lab 2017.

[16] Berman HM, Westbrook J, Feng Z et al. The protein data bank, Nucleic acids research 2000;28:235–242. doi: 10.1093/nar/28.1.235 10592235 PMC102472

[17] Ng PC, Henikoff S. SIFT: Predicting amino acid changes that affect protein function, Nucleic acids research 2003;31:3812–3814. doi: 10.1093/nar/gkg509 12824425 PMC168916

[18] Adzhubei I, Jordan DM, Sunyaev SR. Predicting functional effect of human missense mutations using PolyPhen‐2, Current protocols in human genetics 2013;76:7.20. 21–27.20. 41. doi: 10.1002/0471142905.hg0720s76 23315928 PMC4480630

[19] Choi Y, Chan AP. PROVEAN web server: a tool to predict the functional effect of amino acid substitutions and indels, Bioinformatics 2015;31:2745–2747. doi: 10.1093/bioinformatics/btv195 25851949 PMC4528627

[20] Rentzsch P, Witten D, Cooper GM et al. CADD: predicting the deleteriousness of variants throughout the human genome, Nucleic acids research 2019;47:D886–D894. doi: 10.1093/nar/gky1016 30371827 PMC6323892

[21] Ioannidis NM, Rothstein JH, Pejaver V et al. REVEL: an ensemble method for predicting the pathogenicity of rare missense variants, The American Journal of Human Genetics 2016;99:877–885. doi: 10.1016/j.ajhg.2016.08.016 27666373 PMC5065685

[22] Liu X, Wu C, Li C et al. dbNSFP v3. 0: A one‐stop database of functional predictions and annotations for human nonsynonymous and splice‐site SNVs, Human mutation 2016;37:235–241. doi: 10.1002/humu.22932 26555599 PMC4752381

[23] Ancien F, Pucci F, Godfroid M et al. Prediction and interpretation of deleterious coding variants in terms of protein structural stability, Scientific reports 2018;8:1–11. doi: 10.1038/s41598-017-17765-5 29540703 PMC5852127

[24] Laimer J, Hofer H, Fritz M et al. MAESTRO-multi agent stability prediction upon point mutations, BMC Bioinformatics 2015;16:1–13. doi: 10.1186/s12859-014-0430-y 25885774 PMC4403899

[25] Parthiban V, Gromiha MM, Schomburg D. CUPSAT: prediction of protein stability upon point mutations, Nucleic acids research 2006;34:W239–W242. doi: 10.1093/nar/gkl190 16845001 PMC1538884

[26] Pires DE, Ascher DB, Blundell TL. mCSM: predicting the effects of mutations in proteins using graph-based signatures, Bioinformatics 2014;30:335–342. doi: 10.1093/bioinformatics/btt691 24281696 PMC3904523

[27] Pandurangan AP, Ochoa-Montaño B, Ascher DB et al. SDM: a server for predicting effects of mutations on protein stability, Nucleic Acids Research 2017;45:W229–W235. doi: 10.1093/nar/gkx439 28525590 PMC5793720

[28] Rodrigues CH, Pires DE, Ascher DB. DynaMut: predicting the impact of mutations on protein conformation, flexibility and stability, Nucleic acids research 2018;46:W350–W355. doi: 10.1093/nar/gky300 29718330 PMC6031064

[29] Pejaver V, Urresti J, Lugo-Martinez J et al. MutPred2: inferring the molecular and phenotypic impact of amino acid variants, BioRxiv 2017:134981. 10.1038/s41467-020-19669-x PMC7680112 33219223

[30] Frappier V, Chartier M, Najmanovich RJ. ENCoM server: exploring protein conformational space and the effect of mutations on protein function and stability, Nucleic acids research 2015;43:W395–W400. doi: 10.1093/nar/gkv343 25883149 PMC4489264

[31] Dehouck Y, Grosfils A, Folch B et al. Fast and accurate predictions of protein stability changes upon mutations using statistical potentials and neural networks: PoPMuSiC-2.0, Bioinformatics 2009;25:2537–2543. doi: 10.1093/bioinformatics/btp445 19654118

[32] Pucci F, Bourgeas R, Rooman M. Predicting protein thermal stability changes upon point mutations using statistical potentials: Introducing HoTMuSiC, Scientific reports 2016;6:1–9. doi: 10.1038/s41598-016-0001-8 26988870 PMC4796876

[33] Schrödinger L. Protein preparation wizard, Epik version 2011;2.

[34] DeLano WL. Pymol: An open-source molecular graphics tool, CCP4 Newsletter on protein crystallography 2002;40:82–92.

[35] Shivakumar D, Harder E, Damm W et al. Improving the prediction of absolute solvation free energies using the next generation OPLS force field, Journal of chemical theory and computation 2012;8:2553–2558. doi: 10.1021/ct300203w 26592101

[36] Case DA, Cheatham TE III, Darden T et al. The Amber biomolecular simulation programs, Journal of computational chemistry 2005;26:1668–1688. doi: 10.1002/jcc.20290 16200636 PMC1989667

[37] Maier JA, Martinez C, Kasavajhala K et al. ff14SB: improving the accuracy of protein side chain and backbone parameters from ff99SB, Journal of chemical theory and computation 2015;11:3696–3713. doi: 10.1021/acs.jctc.5b00255 26574453 PMC4821407

[38] Mark P, Nilsson L. Structure and dynamics of the TIP3P, SPC, and SPC/E water models at 298 K, The Journal of Physical Chemistry A 2001;105:9954–9960.

[39] Andersen HC. Rattle: A “velocity” version of the shake algorithm for molecular dynamics calculations, Journal of Computational Physics 1983;52:24–34.

[40] Roe DR, Cheatham TE III. PTRAJ and CPPTRAJ: software for processing and analysis of molecular dynamics trajectory data, Journal of chemical theory and computation 2013;9:3084–3095. doi: 10.1021/ct400341p 26583988

[41] Seifert E. OriginPro 9.1: Scientific Data Analysis and Graphing Software Software Review. ACS Publications, 2014. 10.1021/ci500161d 24702057

[42] Humphrey W, Dalke A, Schulten K. VMD: visual molecular dynamics, Journal of molecular graphics 1996;14:33–38. doi: 10.1016/0263-7855(96)00018-5 8744570

[43] David CC, Jacobs DJ. Principal component analysis: a method for determining the essential dynamics of proteins. Protein dynamics. Springer, 2014, 193–226. 10.1007/978-1-62703-658-0_11 PMC4676806 24061923

[44] Amir M, Mohammad T, Kumar V et al. Structural analysis and conformational dynamics of STN1 gene mutations involved in coat plus syndrome, Frontiers in Molecular Biosciences 2019;6:41. doi: 10.3389/fmolb.2019.00041 31245382 PMC6581698

[45] Naqvi AA, Mohammad T, Hasan GM et al. Advancements in docking and molecular dynamics simulations towards ligand-receptor interactions and structure-function relationships, Current topics in medicinal chemistry 2018;18:1755–1768. doi: 10.2174/1568026618666181025114157 30360721

[46] Papaleo E, Mereghetti P, Fantucci P et al. Free-energy landscape, principal component analysis, and structural clustering to identify representative conformations from molecular dynamics simulations: the myoglobin case, Journal of molecular graphics and modelling 2009;27:889–899. doi: 10.1016/j.jmgm.2009.01.006 19264523

[47] Yates AD, Achuthan P, Akanni W et al. Ensembl 2020, Nucleic acids research 2020;48:D682–D688. doi: 10.1093/nar/gkz966 31691826 PMC7145704

[48] Choudhury A, Mohammad T, Samarth N et al. Structural genomics approach to investigate deleterious impact of nsSNPs in conserved telomere maintenance component 1, Scientific reports 2021;11:1–13. doi: 10.1038/s41598-020-79139-8 33986331 PMC8119478

[49] Habib I, Khan S, Mohammad T et al. Impact of non-synonymous mutations on the structure and function of telomeric repeat binding factor 1, Journal of Biomolecular Structure and Dynamics 2021:1–14. doi: 10.1080/07391102.2021.1922313 33982644

[50] Murphy KP, Freire E. Thermodynamics of structural stability and cooperative folding behavior in proteins, Advances in protein chemistry 1992;43:313–361. doi: 10.1016/s0065-3233(08)60556-2 1442323

[51] Ashkenazy H, Abadi S, Martz E et al. ConSurf 2016: an improved methodology to estimate and visualize evolutionary conservation in macromolecules, Nucleic Acids Research 2016;44:W344–W350. doi: 10.1093/nar/gkw408 27166375 PMC4987940

[52] Berkholz DS, Shapovalov MV, Dunbrack RL Jr et al. Conformation dependence of backbone geometry in proteins, Structure 2009;17:1316–1325. doi: 10.1016/j.str.2009.08.012 19836332 PMC2810841

[53] Saunders WS, Hoyt MA. Kinesin-related proteins required for structural integrity of the mitotic spindle, cell 1992;70:451–458. doi: 10.1016/0092-8674(92)90169-d 1643659

[54] Amir M, Ahamad S, Mohammad T et al. Investigation of conformational dynamics of Tyr89Cys mutation in protection of telomeres 1 gene associated with familial melanoma, Journal of Biomolecular Structure and Dynamics 2019:1–15. doi: 10.1080/07391102.2019.1705186 31847782

[55] Craveur P, Joseph AP, Esque J et al. Protein flexibility in the light of structural alphabets, Frontiers in Molecular Biosciences 2015;2:20. doi: 10.3389/fmolb.2015.00020 26075209 PMC4445325

[56] Khan S, Bjij I, Betz RM et al. Reversible versus irreversible inhibition modes of ERK2: a comparative analysis for ERK2 protein kinase in cancer therapy, Future Medicinal Chemistry 2018;10:1003–1015. doi: 10.4155/fmc-2017-0275 29629569

[57] Lobanov MY, Bogatyreva N, Galzitskaya O. Radius of gyration as an indicator of protein structure compactness, Molecular Biology 2008;42:623–628. 18856071

[58] Durham E, Dorr B, Woetzel N et al. Solvent accessible surface area approximations for rapid and accurate protein structure prediction, Journal of molecular modeling 2009;15:1093–1108. doi: 10.1007/s00894-009-0454-9 19234730 PMC2712621

[59] Myers JK, Pace CN. Hydrogen bonding stabilizes globular proteins, Biophysical Journal 1996;71:2033–2039. doi: 10.1016/S0006-3495(96)79401-8 8889177 PMC1233669

[60] Mohammad T, Siddiqui S, Shamsi A et al. Virtual screening approach to identify high-affinity inhibitors of serum and glucocorticoid-regulated kinase 1 among bioactive natural products: Combined molecular docking and simulation studies, Molecules 2020;25:823. doi: 10.3390/molecules25040823 32070031 PMC7070812

[61] Hattori K, Sakata-Yanagimoto M, Okoshi Y et al. MYD88 (L265P) mutation is associated with an unfavourable outcome of primary central nervous system lymphoma, British journal of haematology 2017;177:492–494. doi: 10.1111/bjh.14080 27161435

[62] White BS, Lanc I, O’Neal J et al. A multiple myeloma-specific capture sequencing platform discovers novel translocations and frequent, risk-associated point mutations in IGLL5, Blood cancer journal 2018;8:1–10. doi: 10.1038/s41408-017-0043-6 29563506 PMC5862875

