# Supplementary material for: Investigating single amino acid substitutions in PIM1 kinase: A structural genomics approach
Source: PLoS One. 2021 Oct 22;16(10):e0258929. doi: 10.1371/journal.pone.0258929 (PMC8535467; doi:10.1371/journal.pone.0258929)
Supplement: S1 Fig — Structural snapshots of PIM1 (A) WT, (B) F147C, and (C) W109C at an interval of 50 ns from 0 to 200 ns of simulation. Corresponding panels show the RMSD of structural alignment of PIM1 at different times. Structures were drawn using PyMOL (https://pymol.org/2). (DOCX) [file pone.0258929.s001.docx]

**
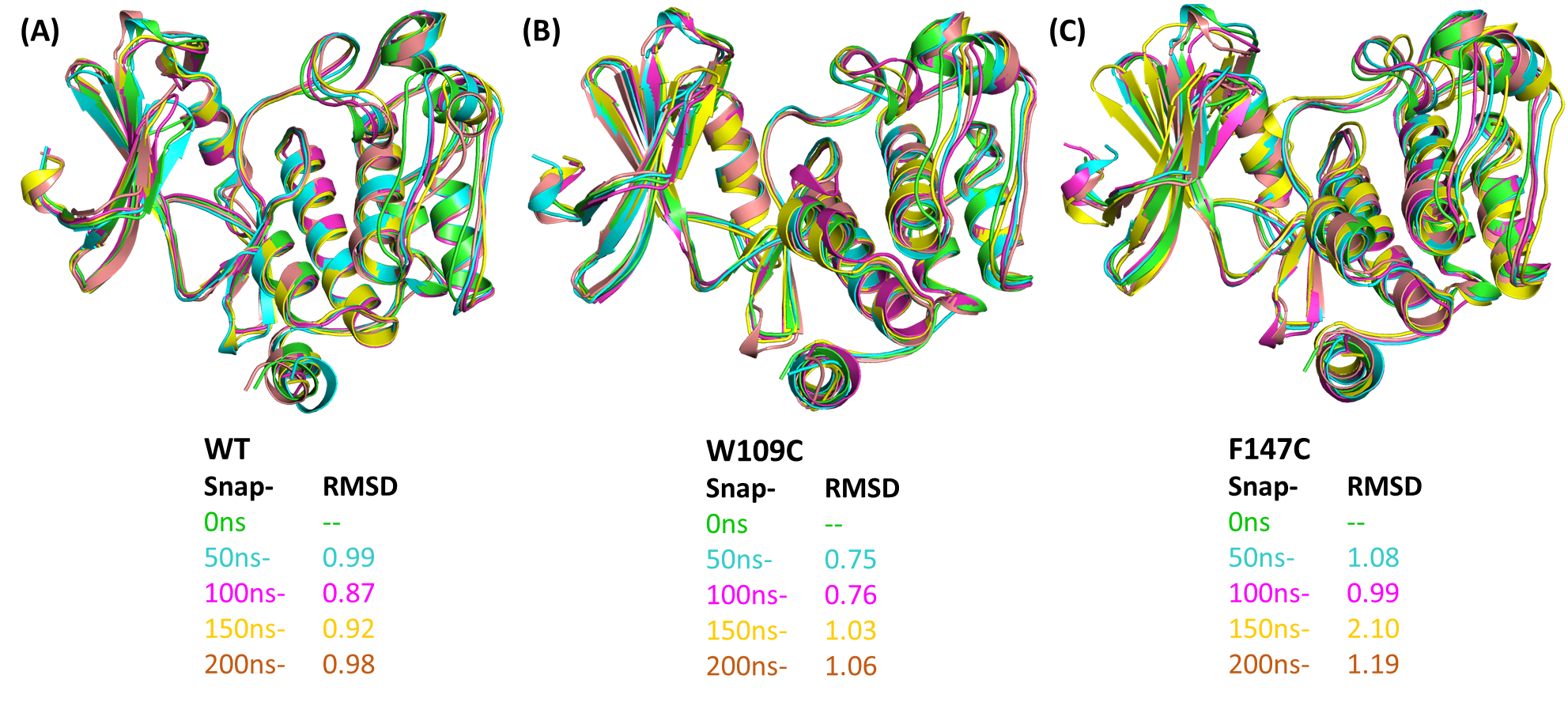
**

**Figure S1:** Structural snapshots of PIM1 (**A**) WT, (**B**) F147C, and (**C**) W109C at an interval of 50 ns from 0 to 200 ns of simulation. Corresponding panels show the RMSD of structural alignment of PIM1 at different times. Structures were drawn using PyMOL (https://pymol.org/2).
