# Supplementary material for: Investigating single amino acid substitutions in PIM1 kinase: A structural genomics approach
Source: PLoS One. 2021 Oct 22;16(10):e0258929. doi: 10.1371/journal.pone.0258929 (PMC8535467; doi:10.1371/journal.pone.0258929)
Supplement: S2 Table — (DOCX) [file pone.0258929.s003.docx]

**Table S2**. Structure based prediction of mutations associated with PIM1 kinase domain.

| **Variant ID** | **Variant** | **Predictor** | | | | | | | | | | |
| --- | --- | --- | --- | --- | --- | --- | --- | --- | --- | --- | --- | --- |
|  |  | **Maestro** | **CUPSAT** | **mCSM** | **SDM** | **DUET** | **Dynamut** | **DynaMut2** | **MutPred2** | **ENCoM** | **PoPMuSiC** | **HoTMuSiC** |
| rs761386793 | Y38C | Destabilizing | Destabilizing | Destabilizing | Destabilizing | Destabilizing | Destabilizing | Destabilizing | Destabilizing | Destabilizing | Destabilizing | Destabilizing |
| rs1387437361 | Q39H | Stabilizing | Stabilizing | Destabilizing | Stabilizing | Stabilizing | Destabilizing | Stabilizing | Stabilizing | Destabilizing | Destabilizing | Stabilizing |
| rs1387437361 | Q39R | Destabilizing | Destabilizing | Stabilizing | Stabilizing | Stabilizing | Destabilizing | Stabilizing | Stabilizing | Stabilizing | Destabilizing | Destabilizing |
| rs767621897 | Q39L | Destabilizing | Stabilizing | Stabilizing | Stabilizing | Stabilizing | Destabilizing | Stabilizing | Stabilizing | Destabilizing | Destabilizing | Destabilizing |
| rs1167511555 | V40L | Stabilizing | Destabilizing | Destabilizing | Destabilizing | Destabilizing | Stabilizing | Destabilizing | Stabilizing | Stabilizing | Destabilizing | Destabilizing |
| rs750381293 | V40G | Destabilizing | Destabilizing | Destabilizing | Destabilizing | Destabilizing | Destabilizing | Destabilizing | Less Destabilizing | Destabilizing | Destabilizing | Destabilizing |
| rs766108688 | P42R | Destabilizing | Destabilizing | Stabilizing | Stabilizing | Stabilizing | Stabilizing | Stabilizing | Stabilizing | Destabilizing | Destabilizing | Destabilizing |
| rs1337351112 | G45S | Stabilizing | Stabilizing | Destabilizing | Destabilizing | Destabilizing | Destabilizing | Destabilizing | Destabilizing | Destabilizing | Destabilizing | Stabilizing |
| rs758586647 | G48D | Destabilizing | Stabilizing | Destabilizing | Stabilizing | Destabilizing | Destabilizing | Destabilizing | Less Destabilizing | Destabilizing | Stabilizing | Stabilizing |
| rs778032371 | G50D | Stabilizing | Stabilizing | Stabilizing | Destabilizing | Stabilizing | Stabilizing | Stabilizing | Destabilizing | Stabilizing | Destabilizing | Destabilizing |
| rs778032371 | G50A | Stabilizing | Stabilizing | Destabilizing | Destabilizing | Destabilizing | Stabilizing | Destabilizing | Destabilizing | Stabilizing | Destabilizing | Destabilizing |
| rs551680157 | S51L | Stabilizing | Stabilizing | Stabilizing | Stabilizing | Stabilizing | Stabilizing | Destabilizing | Less Destabilizing | Stabilizing | Stabilizing | Stabilizing |
| rs551680157 | S51W | Stabilizing | Stabilizing | Destabilizing | Stabilizing | Destabilizing | Stabilizing | Destabilizing | Stabilizing | Stabilizing | Destabilizing | Destabilizing |
| rs781178342 | S54T | Stabilizing | Stabilizing | Destabilizing | Stabilizing | Destabilizing | Stabilizing | Stabilizing | Stabilizing | Stabilizing | Destabilizing | Destabilizing |
| rs1222607867 | R57C | Destabilizing | Destabilizing | Destabilizing | Stabilizing | Destabilizing | Destabilizing | Destabilizing | Less Destabilizing | Destabilizing | Destabilizing | Destabilizing |
| rs1323508876 | R57H | Destabilizing | Destabilizing | Destabilizing | Stabilizing | Destabilizing | Destabilizing | Destabilizing | Stabilizing | Stabilizing | Destabilizing | Destabilizing |
| rs896496531 | V58F | Stabilizing | Stabilizing | Destabilizing | Stabilizing | Destabilizing | Destabilizing | Destabilizing | Stabilizing | Destabilizing | Destabilizing | Destabilizing |
| rs896496531 | V58I | Stabilizing | Destabilizing | Destabilizing | Stabilizing | Destabilizing | Stabilizing | Destabilizing | Stabilizing | Stabilizing | Destabilizing | Destabilizing |
| rs936800231 | S59C | Destabilizing | Stabilizing | Destabilizing | Stabilizing | Destabilizing | Stabilizing | Destabilizing | Stabilizing | Stabilizing | Destabilizing | Destabilizing |
| rs1318584789 | D60N | Destabilizing | Destabilizing | Stabilizing | Destabilizing | Destabilizing | Destabilizing | Stabilizing | Less Destabilizing | Stabilizing | Stabilizing | Destabilizing |
| rs1279827128 | P63L | Stabilizing | Destabilizing | Destabilizing | Stabilizing | Stabilizing | Stabilizing | Destabilizing | Stabilizing | Stabilizing | Destabilizing | Destabilizing |
| rs536789134 | H68Q | Destabilizing | Stabilizing | Destabilizing | Destabilizing | Destabilizing | Destabilizing | Destabilizing | Stabilizing | Destabilizing | Destabilizing | Destabilizing |
| rs750555644 | H68R | Destabilizing | Stabilizing | Destabilizing | Destabilizing | Destabilizing | Stabilizing | Destabilizing | Stabilizing | Stabilizing | Destabilizing | Destabilizing |
| rs780494083 | V69M | Stabilizing | Stabilizing | Destabilizing | Destabilizing | Destabilizing | Destabilizing | Destabilizing | Less Destabilizing | Stabilizing | Destabilizing | Destabilizing |
| rs780494083 | V69L | Stabilizing | Stabilizing | Destabilizing | Destabilizing | Destabilizing | Stabilizing | Destabilizing | Less Destabilizing | Stabilizing | Destabilizing | Destabilizing |
| rs1300349300 | E70G | Destabilizing | Destabilizing | Destabilizing | Stabilizing | Destabilizing | Stabilizing | Destabilizing | Stabilizing | Stabilizing | Stabilizing | Destabilizing |
| rs576745819 | E70K | Destabilizing | Destabilizing | Destabilizing | Destabilizing | Stabilizing | Stabilizing | Destabilizing | Stabilizing | Stabilizing | Destabilizing | Destabilizing |
| rs1460977599 | D72Y | Destabilizing | Destabilizing | Stabilizing | Stabilizing | Stabilizing | Stabilizing | Stabilizing | Less Destabilizing | Stabilizing | Destabilizing | Stabilizing |
| rs373869744 | D72G | Stabilizing | Destabilizing | Destabilizing | Stabilizing | Destabilizing | Stabilizing | Destabilizing | Less Destabilizing | Stabilizing | Destabilizing | Destabilizing |
| rs373869744 | D72V | Stabilizing | Stabilizing | Stabilizing | Stabilizing | Stabilizing | Destabilizing | Stabilizing | Less Destabilizing | Stabilizing | Destabilizing | Destabilizing |
| rs748490577 | R73W | Stabilizing | Destabilizing | Destabilizing | Destabilizing | Destabilizing | Destabilizing | Destabilizing | Stabilizing | Stabilizing | Destabilizing | Destabilizing |
| rs1308265632 | I74F | Stabilizing | Destabilizing | Destabilizing | Destabilizing | Destabilizing | Stabilizing | Destabilizing | Less Destabilizing | Stabilizing | Destabilizing | Destabilizing |
| rs771898144 | D76H | Destabilizing | Stabilizing | Destabilizing | Stabilizing | Stabilizing | Stabilizing | Stabilizing | Stabilizing | Stabilizing | Stabilizing | Stabilizing |
| rs771898144 | D76E | Destabilizing | Stabilizing | Destabilizing | Stabilizing | Destabilizing | Stabilizing | Stabilizing | Less Destabilizing | Stabilizing | Stabilizing | Stabilizing |
| rs1244502757 | D76Y | Destabilizing | Stabilizing | Destabilizing | Stabilizing | Stabilizing | Stabilizing | Destabilizing | Stabilizing | Stabilizing | Destabilizing | Destabilizing |
| rs149210226 | N82I | Destabilizing | Stabilizing | Stabilizing | Stabilizing | Stabilizing | Destabilizing | Stabilizing | Stabilizing | Destabilizing | Stabilizing | Destabilizing |
| rs367638496 | N82T | Destabilizing | Stabilizing | Stabilizing | Destabilizing | Stabilizing | Destabilizing | Stabilizing | Stabilizing | Destabilizing | Stabilizing | Destabilizing |
| rs367638496 | N82S | Destabilizing | Stabilizing | Stabilizing | Destabilizing | Stabilizing | Stabilizing | Stabilizing | Stabilizing | Destabilizing | Stabilizing | Destabilizing |
| rs367638496 | N82D | Destabilizing | Destabilizing | Stabilizing | Destabilizing | Stabilizing | Destabilizing | Stabilizing | Stabilizing | Destabilizing | Destabilizing | Destabilizing |
| rs745445786 | T84I | Destabilizing | Destabilizing | Destabilizing | Stabilizing | Stabilizing | Stabilizing | Destabilizing | Stabilizing | Stabilizing | Destabilizing | Destabilizing |
| rs755619965 | P87A | Destabilizing | Destabilizing | Destabilizing | Stabilizing | Destabilizing | Stabilizing | Destabilizing | Less Destabilizing | Destabilizing | Destabilizing | Destabilizing |
| rs1212225125 | M88I | Destabilizing | Stabilizing | Destabilizing | Stabilizing | Destabilizing | Stabilizing | Destabilizing | Stabilizing | Destabilizing | Destabilizing | Destabilizing |
| rs779472533 | M88T | Destabilizing | Destabilizing | Destabilizing | Destabilizing | Destabilizing | Destabilizing | Destabilizing | Less Destabilizing | Destabilizing | Destabilizing | Destabilizing |
| rs1561983108 | M88V | Destabilizing | Destabilizing | Destabilizing | Stabilizing | Destabilizing | Destabilizing | Destabilizing | Stabilizing | Destabilizing | Destabilizing | Destabilizing |
| rs1240119838 | V91I | Destabilizing | Stabilizing | Destabilizing | Destabilizing | Destabilizing | Destabilizing | Destabilizing | Stabilizing | Stabilizing | Destabilizing | Destabilizing |
| rs548710452 | L93Q | Destabilizing | Destabilizing | Destabilizing | Destabilizing | Destabilizing | Destabilizing | Destabilizing | Less Destabilizing | Destabilizing | Destabilizing | Destabilizing |
| rs747872030 | L93V | Destabilizing | Stabilizing | Destabilizing | Destabilizing | Destabilizing | Destabilizing | Destabilizing | Destabilizing | Destabilizing | Destabilizing | Destabilizing |
| rs771241383 | K94R | Stabilizing | Destabilizing | Destabilizing | Stabilizing | Destabilizing | Stabilizing | Destabilizing | Stabilizing | Stabilizing | Stabilizing | Destabilizing |
| rs771241383 | K94M | Stabilizing | Destabilizing | Destabilizing | Stabilizing | Stabilizing | Destabilizing | Stabilizing | Stabilizing | Destabilizing | Destabilizing | Destabilizing |
| rs776712611 | K95R | Stabilizing | Destabilizing | Destabilizing | Stabilizing | Destabilizing | Destabilizing | Destabilizing | Stabilizing | Destabilizing | Stabilizing | Destabilizing |
| rs1052690711 | V96L | Destabilizing | Stabilizing | Destabilizing | Stabilizing | Destabilizing | Stabilizing | Destabilizing | Less Destabilizing | Stabilizing | Destabilizing | Destabilizing |
| rs562319987 | S97N | Destabilizing | Destabilizing | Destabilizing | Stabilizing | Destabilizing | Destabilizing | Destabilizing | Stabilizing | Destabilizing | Destabilizing | Destabilizing |
| rs531504155 | S101F | Stabilizing | Destabilizing | Destabilizing | Stabilizing | Destabilizing | Stabilizing | Destabilizing | Less Destabilizing | Stabilizing | Stabilizing | Destabilizing |
| rs1256711166 | V103I | Stabilizing | Destabilizing | Destabilizing | Destabilizing | Destabilizing | Stabilizing | Destabilizing | Stabilizing | Stabilizing | Destabilizing | Destabilizing |
| rs1343874841 | L106P | Destabilizing | Stabilizing | Destabilizing | Destabilizing | Destabilizing | Destabilizing | Destabilizing | Less Destabilizing | Destabilizing | Destabilizing | Destabilizing |
| rs764662779 | L106F | Destabilizing | Stabilizing | Destabilizing | Destabilizing | Destabilizing | Stabilizing | Destabilizing | Destabilizing | Stabilizing | Destabilizing | Destabilizing |
| rs1283986952 | W109C | Destabilizing | Destabilizing | Destabilizing | Destabilizing | Destabilizing | Destabilizing | Destabilizing | Destabilizing | Destabilizing | Destabilizing | Destabilizing |
| rs757552417 | F110L | Stabilizing | Stabilizing | Destabilizing | Stabilizing | Destabilizing | Destabilizing | Destabilizing | Less Destabilizing | Destabilizing | Destabilizing | Destabilizing |
| rs767270626 | E111Q | Stabilizing | Destabilizing | Destabilizing | Destabilizing | Destabilizing | Destabilizing | Destabilizing | Destabilizing | Destabilizing | Stabilizing | Destabilizing |
| rs750053312 | R112G | Destabilizing | Destabilizing | Destabilizing | Stabilizing | Destabilizing | Destabilizing | Destabilizing | Less Destabilizing | Destabilizing | Destabilizing | Destabilizing |
| rs779562379 | D114H | Destabilizing | Stabilizing | Destabilizing | Stabilizing | Destabilizing | Destabilizing | Destabilizing | Destabilizing | Stabilizing | Destabilizing | Destabilizing |
| rs748757329 | D114E | Destabilizing | Stabilizing | Destabilizing | Stabilizing | Stabilizing | Destabilizing | Destabilizing | Less Destabilizing | Stabilizing | Destabilizing | Destabilizing |
| rs754950017 | S115R | Stabilizing | Stabilizing | Destabilizing | Stabilizing | Destabilizing | Stabilizing | Destabilizing | Less Destabilizing | Destabilizing | Destabilizing | Destabilizing |
| rs778700298 | F116Y | Destabilizing | Destabilizing | Destabilizing | Destabilizing | Destabilizing | Stabilizing | Destabilizing | Destabilizing | Stabilizing | Destabilizing | Destabilizing |
| rs1489714428 | F116V | Stabilizing | Destabilizing | Destabilizing | Destabilizing | Destabilizing | Destabilizing | Destabilizing | Less Destabilizing | Destabilizing | Destabilizing | Destabilizing |
| rs746110686 | L120R | Destabilizing | Stabilizing | Destabilizing | Destabilizing | Destabilizing | Destabilizing | Destabilizing | Destabilizing | Stabilizing | Destabilizing | Destabilizing |
| rs770097476 | E121D | Stabilizing | Destabilizing | Destabilizing | Destabilizing | Destabilizing | Destabilizing | Destabilizing | Destabilizing | Destabilizing | Destabilizing | Destabilizing |
| rs1193992710 | R122K | Destabilizing | Destabilizing | Destabilizing | Destabilizing | Destabilizing | Destabilizing | Destabilizing | Less Destabilizing | Destabilizing | Destabilizing | Destabilizing |
| rs35760989 | E124Q | Destabilizing | Destabilizing | Destabilizing | Destabilizing | Destabilizing | Destabilizing | Destabilizing | Stabilizing | Stabilizing | Stabilizing | Destabilizing |
| rs760353228 | F132L | Destabilizing | Destabilizing | Destabilizing | Destabilizing | Destabilizing | Destabilizing | Stabilizing | Destabilizing | Destabilizing | Destabilizing | Destabilizing |
| rs200523275 | E135K | Stabilizing | Destabilizing | Stabilizing | Destabilizing | Stabilizing | Destabilizing | Stabilizing | Less Destabilizing | Destabilizing | Stabilizing | Destabilizing |
| rs753402545 | R136K | Stabilizing | Stabilizing | Destabilizing | Destabilizing | Destabilizing | Destabilizing | Stabilizing | Stabilizing | Destabilizing | Destabilizing | Destabilizing |
| rs754478439 | Q140K | Destabilizing | Stabilizing | Destabilizing | Stabilizing | Stabilizing | Destabilizing | Destabilizing | Less Destabilizing | Destabilizing | Stabilizing | Destabilizing |
| rs33989191 | E142D | Destabilizing | Destabilizing | Destabilizing | Destabilizing | Destabilizing | Destabilizing | Destabilizing | Stabilizing | Destabilizing | Destabilizing | Destabilizing |
| rs1452774267 | A144D | Destabilizing | Destabilizing | Destabilizing | Destabilizing | Destabilizing | Destabilizing | Destabilizing | Destabilizing | Stabilizing | Destabilizing | Destabilizing |
| rs1231875195 | F147C | Destabilizing | Destabilizing | Destabilizing | Destabilizing | Destabilizing | Destabilizing | Destabilizing | Destabilizing | Destabilizing | Destabilizing | Destabilizing |
| rs758303054 | F148L | Destabilizing | Destabilizing | Destabilizing | Destabilizing | Destabilizing | Destabilizing | Stabilizing | Destabilizing | Destabilizing | Destabilizing | Destabilizing |
| rs770187449 | V155L | Destabilizing | Stabilizing | Destabilizing | Stabilizing | Destabilizing | Destabilizing | Destabilizing | Less Destabilizing | Stabilizing | Destabilizing | Destabilizing |
| rs894956820 | R156Q | Stabilizing | Destabilizing | Destabilizing | Destabilizing | Destabilizing | Destabilizing | Destabilizing | Stabilizing | Destabilizing | Destabilizing | Destabilizing |
| rs141143854 | H157Y | Destabilizing | Destabilizing | Stabilizing | Destabilizing | Stabilizing | Stabilizing | Stabilizing | Less Destabilizing | Stabilizing | Destabilizing | Destabilizing |
| rs200536541 | H157P | Destabilizing | Destabilizing | Destabilizing | Destabilizing | Destabilizing | Destabilizing | Destabilizing | Destabilizing | Destabilizing | Destabilizing | Destabilizing |
| rs768626728 | H159Q | Destabilizing | Destabilizing | Destabilizing | Stabilizing | Destabilizing | Destabilizing | Destabilizing | Less Destabilizing | Destabilizing | Destabilizing | Stabilizing |
| rs774435785 | N160H | Destabilizing | Stabilizing | Destabilizing | Stabilizing | Destabilizing | Stabilizing | Destabilizing | Stabilizing | Stabilizing | Destabilizing | Destabilizing |
| rs774435785 | N160S | Stabilizing | Stabilizing | Destabilizing | Destabilizing | Stabilizing | Stabilizing | Destabilizing | Stabilizing | Destabilizing | Destabilizing | Destabilizing |
| rs762249073 | N160D | Stabilizing | Destabilizing | Destabilizing | Stabilizing | Stabilizing | Destabilizing | Stabilizing | Stabilizing | Destabilizing | Stabilizing | Destabilizing |
| rs761032394 | G162R | Destabilizing | Destabilizing | Destabilizing | Destabilizing | Destabilizing | Destabilizing | Destabilizing | Destabilizing | Stabilizing | Destabilizing | Destabilizing |
| rs772592158 | R166H | Destabilizing | Destabilizing | Destabilizing | Destabilizing | Destabilizing | Destabilizing | Destabilizing | Destabilizing | Destabilizing | Destabilizing | Destabilizing |
| rs773608051 | R166C | Destabilizing | Stabilizing | Destabilizing | Destabilizing | Destabilizing | Destabilizing | Destabilizing | Destabilizing | Destabilizing | Destabilizing | Destabilizing |
| rs1201030656 | D176N | Destabilizing | Stabilizing | Destabilizing | Destabilizing | Destabilizing | Stabilizing | Destabilizing | Less Destabilizing | Destabilizing | Destabilizing | Destabilizing |
| rs764675670 | L177P | Destabilizing | Destabilizing | Destabilizing | Destabilizing | Destabilizing | Destabilizing | Destabilizing | Destabilizing | Destabilizing | Destabilizing | Destabilizing |
| rs752207702 | N178S | Destabilizing | Destabilizing | Destabilizing | Destabilizing | Destabilizing | Destabilizing | Stabilizing | Stabilizing | Destabilizing | Stabilizing | Destabilizing |
| rs200495767 | L184V | Destabilizing | Destabilizing | Destabilizing | Destabilizing | Destabilizing | Destabilizing | Destabilizing | Destabilizing | Destabilizing | Destabilizing | Destabilizing |
| rs1467271687 | G188A | Destabilizing | Stabilizing | Destabilizing | Stabilizing | Destabilizing | Stabilizing | Destabilizing | Destabilizing | Stabilizing | Destabilizing | Destabilizing |
| rs1451904001 | K194E | Destabilizing | Stabilizing | Stabilizing | Destabilizing | Stabilizing | Stabilizing | Destabilizing | Less Destabilizing | Destabilizing | Destabilizing | Destabilizing |
| rs1274563992 | T196A | Destabilizing | Destabilizing | Destabilizing | Stabilizing | Destabilizing | Stabilizing | Destabilizing | Less Destabilizing | Destabilizing | Destabilizing | Destabilizing |
| rs137884665 | V197F | Destabilizing | Stabilizing | Destabilizing | Destabilizing | Destabilizing | Stabilizing | Destabilizing | Less Destabilizing | Stabilizing | Destabilizing | Stabilizing |
| rs778892108 | F201L | Destabilizing | Destabilizing | Destabilizing | Destabilizing | Destabilizing | Destabilizing | Destabilizing | Destabilizing | Destabilizing | Destabilizing | Destabilizing |
| rs1583399145 | T204P | Stabilizing | Stabilizing | Destabilizing | Stabilizing | Destabilizing | Destabilizing | Destabilizing | Destabilizing | Destabilizing | Destabilizing | Stabilizing |
| rs199791472 | V206E | Destabilizing | Destabilizing | Destabilizing | Destabilizing | Destabilizing | Destabilizing | Destabilizing | Destabilizing | Destabilizing | Destabilizing | Stabilizing |
| rs1255159914 | Y207C | Destabilizing | Stabilizing | Destabilizing | Destabilizing | Destabilizing | Destabilizing | Destabilizing | Destabilizing | Destabilizing | Destabilizing | Destabilizing |
| rs1183187519 | S208I | Stabilizing | Destabilizing | Stabilizing | Stabilizing | Stabilizing | Stabilizing | Stabilizing | Destabilizing | Stabilizing | Destabilizing | Destabilizing |
| rs1259247608 | P209L | Stabilizing | Destabilizing | Destabilizing | Stabilizing | Destabilizing | Stabilizing | Destabilizing | Destabilizing | Stabilizing | Destabilizing | Destabilizing |
| rs1038105494 | I213V | Destabilizing | Destabilizing | Destabilizing | Destabilizing | Destabilizing | Destabilizing | Destabilizing | Stabilizing | Destabilizing | Destabilizing | Destabilizing |
| rs752976281 | R214H | Destabilizing | Stabilizing | Destabilizing | Destabilizing | Destabilizing | Stabilizing | Stabilizing | Less Destabilizing | Stabilizing | Destabilizing | Destabilizing |
| rs1442496121 | R214C | Destabilizing | Destabilizing | Destabilizing | Destabilizing | Destabilizing | Stabilizing | Destabilizing | Stabilizing | Stabilizing | Destabilizing | Destabilizing |
| rs1468671308 | Y215H | Destabilizing | Stabilizing | Destabilizing | Destabilizing | Stabilizing | Destabilizing | Stabilizing | Less Destabilizing | Destabilizing | Stabilizing | Destabilizing |
| rs1176016480 | R217H | Destabilizing | Destabilizing | Destabilizing | Stabilizing | Destabilizing | Destabilizing | Stabilizing | Destabilizing | Destabilizing | Destabilizing | Destabilizing |
| rs1244024334 | R217C | Destabilizing | Destabilizing | Destabilizing | Destabilizing | Destabilizing | Destabilizing | Destabilizing | Destabilizing | Destabilizing | Destabilizing | Destabilizing |
| rs1467915137 | R221M | Stabilizing | Stabilizing | Destabilizing | Stabilizing | Destabilizing | Destabilizing | Destabilizing | Destabilizing | Destabilizing | Destabilizing | Destabilizing |
| rs1583399226 | S222A | Destabilizing | Destabilizing | Destabilizing | Stabilizing | Stabilizing | Stabilizing | Destabilizing | Less Destabilizing | Destabilizing | Destabilizing | Stabilizing |
| rs758565911 | V225I | Destabilizing | Stabilizing | Destabilizing | Destabilizing | Destabilizing | Stabilizing | Destabilizing | Less Destabilizing | Destabilizing | Destabilizing | Destabilizing |
| rs1583399253 | V225G | Destabilizing | Destabilizing | Destabilizing | Destabilizing | Destabilizing | Destabilizing | Destabilizing | Destabilizing | Destabilizing | Destabilizing | Destabilizing |
| rs1583399259 | S227A | Destabilizing | Stabilizing | Destabilizing | Stabilizing | Destabilizing | Stabilizing | Destabilizing | Less Destabilizing | Destabilizing | Destabilizing | Destabilizing |
| rs760297171 | D234N | Destabilizing | Destabilizing | Destabilizing | Destabilizing | Destabilizing | Destabilizing | Destabilizing | Less Destabilizing | Destabilizing | Destabilizing | Destabilizing |
| rs1583399274 | V236G | Destabilizing | Destabilizing | Destabilizing | Destabilizing | Destabilizing | Destabilizing | Destabilizing | Destabilizing | Destabilizing | Destabilizing | Destabilizing |
| rs1389606669 | D239G | Destabilizing | Stabilizing | Destabilizing | Stabilizing | Destabilizing | Stabilizing | Destabilizing | Destabilizing | Destabilizing | Destabilizing | Destabilizing |
| rs757806837 | I240T | Destabilizing | Stabilizing | Destabilizing | Destabilizing | Destabilizing | Destabilizing | Destabilizing | Less Destabilizing | Destabilizing | Destabilizing | Destabilizing |
| rs371562493 | H244R | Destabilizing | Destabilizing | Destabilizing | Destabilizing | Destabilizing | Stabilizing | Stabilizing | Stabilizing | Stabilizing | Destabilizing | Destabilizing |
| rs773910971 | E246K | Destabilizing | Destabilizing | Destabilizing | Destabilizing | Destabilizing | Stabilizing | Destabilizing | Less Destabilizing | Stabilizing | Destabilizing | Destabilizing |
| rs1583399317 | I249M | Destabilizing | Stabilizing | Destabilizing | Destabilizing | Destabilizing | Destabilizing | Stabilizing | Stabilizing | Destabilizing | Destabilizing | Destabilizing |
| rs1480836301 | G251S | Destabilizing | Destabilizing | Destabilizing | Destabilizing | Destabilizing | Stabilizing | Destabilizing | Stabilizing | Stabilizing | Destabilizing | Destabilizing |
| rs1053377617 | F254Y | Destabilizing | Stabilizing | Destabilizing | Destabilizing | Destabilizing | Stabilizing | Stabilizing | Stabilizing | Stabilizing | Destabilizing | Destabilizing |
| rs1053377617 | F254V | Destabilizing | Stabilizing | Destabilizing | Stabilizing | Destabilizing | Stabilizing | Stabilizing | Stabilizing | Destabilizing | Destabilizing | Destabilizing |
| rs1487749896 | F254L | Destabilizing | Stabilizing | Destabilizing | Stabilizing | Destabilizing | Destabilizing | Destabilizing | Stabilizing | Destabilizing | Stabilizing | Destabilizing |
| rs1264658833 | Q257R | Destabilizing | Destabilizing | Stabilizing | Stabilizing | Stabilizing | Destabilizing | Stabilizing | Stabilizing | Destabilizing | Destabilizing | Destabilizing |
| rs1292506227 | R258S | Destabilizing | Destabilizing | Destabilizing | Destabilizing | Destabilizing | Destabilizing | Destabilizing | Less Destabilizing | Destabilizing | Destabilizing | Destabilizing |
| rs1480448252 | V259I | Destabilizing | Destabilizing | Destabilizing | Destabilizing | Destabilizing | Stabilizing | Destabilizing | Stabilizing | Stabilizing | Destabilizing | Destabilizing |
| rs748993573 | S260A | Destabilizing | Destabilizing | Destabilizing | Stabilizing | Destabilizing | Destabilizing | Destabilizing | Destabilizing | Destabilizing | Destabilizing | Destabilizing |
| rs893105837 | S260F | Destabilizing | Destabilizing | Destabilizing | Stabilizing | Destabilizing | Destabilizing | Destabilizing | Destabilizing | Stabilizing | Destabilizing | Destabilizing |
| rs1426492223 | S261L | Destabilizing | Destabilizing | Destabilizing | Stabilizing | Stabilizing | Stabilizing | Destabilizing | Less Destabilizing | Stabilizing | Destabilizing | Destabilizing |
| rs770738366 | A272S | Destabilizing | Destabilizing | Destabilizing | Destabilizing | Destabilizing | Stabilizing | Destabilizing | Stabilizing | Stabilizing | Destabilizing | Stabilizing |
| rs1181689554 | D277N | Destabilizing | Destabilizing | Destabilizing | Stabilizing | Destabilizing | Stabilizing | Destabilizing | Less Destabilizing | Destabilizing | Stabilizing | Destabilizing |
| rs1451627047 | T280A | Destabilizing | Stabilizing | Destabilizing | Stabilizing | Destabilizing | Stabilizing | Destabilizing | Stabilizing | Stabilizing | Destabilizing | Destabilizing |
| rs1356811164 | F281V | Destabilizing | Destabilizing | Destabilizing | Destabilizing | Destabilizing | Destabilizing | Destabilizing | Less Destabilizing | Destabilizing | Destabilizing | Destabilizing |
| rs148258857 | F281L | Destabilizing | Destabilizing | Destabilizing | Destabilizing | Destabilizing | Destabilizing | Destabilizing | Less Destabilizing | Destabilizing | Destabilizing | Destabilizing |
| rs761967890 | E282V | Destabilizing | Destabilizing | Stabilizing | Destabilizing | Destabilizing | Destabilizing | Stabilizing | Stabilizing | Destabilizing | Destabilizing | Destabilizing |
| rs141695323 | E282K | Destabilizing | Destabilizing | Stabilizing | Destabilizing | Stabilizing | Stabilizing | Destabilizing | Stabilizing | Destabilizing | Destabilizing | Destabilizing |
| rs141695323 | E282A | Destabilizing | Destabilizing | Destabilizing | Destabilizing | Destabilizing | Destabilizing | Stabilizing | Less Destabilizing | Destabilizing | Destabilizing | Destabilizing |
| rs1583399912 | N286T | Destabilizing | Destabilizing | Destabilizing | Destabilizing | Destabilizing | Destabilizing | Stabilizing | Stabilizing | Destabilizing | Stabilizing | Destabilizing |
| rs750431197 | P288S | Stabilizing | Destabilizing | Destabilizing | Stabilizing | Destabilizing | Stabilizing | Destabilizing | Stabilizing | Stabilizing | Destabilizing | Destabilizing |
| rs1238113576 | P288R | Stabilizing | Destabilizing | Destabilizing | Stabilizing | Stabilizing | Stabilizing | Destabilizing | Stabilizing | Stabilizing | Destabilizing | Destabilizing |
| rs1210588080 | M290V | Destabilizing | Destabilizing | Destabilizing | Destabilizing | Destabilizing | Destabilizing | Destabilizing | Stabilizing | Destabilizing | Destabilizing | Destabilizing |
